# Complete mitochondrial genomics reveals phylogenetic relationships and mitogenomic features in six ectomycorrhizal *Russula* species

**DOI:** 10.3389/fmicb.2026.1865163

**Published:** 2026-07-10

**Authors:** Xianyi Wang, Huangxue Luo, Qun Luo, Guangyin Xu, Yaping Wang, Yue Zhang, Hongmei Liu

**Affiliations:** 1 Engineering Research Center of Health Medicine Biotechnology of Institution of Higher Education of Department of Education of Guizhou Province, Guizhou Medical University, Guiyang, China; 2 Engineering Research Center of Medical Biotechnology, School of Biology and Engineering, Guizhou Medical University, Guiyang, China; 3 The High Efficacy Application of Natural Medicinal Resources Engineering Center of Guizhou Province (The Key Laboratory of Optimal Utilization of Natural Medicine Resources), School of Pharmaceutical Sciences, Guizhou Medical University, Guiyang, China; 4 Laboratory Animal Center of Guizhou Medical University, Guiyang, China

**Keywords:** ectomycorrhiza, fungi, mitochondrial evolution, mitogenome, *Russula*

## Abstract

**Introduction:**

*Russula* (Russulaceae, Basidiomycota) is a widely distributed genus of ectomycorrhizal fungi.

**Methods:**

In this study, we assembled and annotated the complete mitochondrial genomes (mitogenomes) of six *Russula* species.

**Results:**

The mitogenomes ranged from 41,017 bp (*R. hookeri*) to 57,170 bp (*R*. aff. *cessans*). This size variation appears to be partly influenced by intron content, as the smallest genome (*R. hookeri*) lacks introns in the *cox1* gene. All mitogenomes contained 15 core protein-coding genes (PCGs) (*atp6*, *atp8*, *atp9*, *cob*, *cox1*-*cox3*, *nad1*-*nad6*, *nad4L*, and *rps3*), two rRNA genes, and 25–27 tRNA genes, with the largest intergenic spacer consistently located between *rrnL* and *nad6*. Codon usage showed a strong bias toward A/U-ending codons, with UUA (Leu) and AGA (Arg) exhibiting the highest relative synonymous codon usage (RSCU) values. Notably, *trnM* was newly annotated in the *trnE*-*trnL* region across all six species. Selective pressure analysis revealed that 14 of the 15 core PCGs were under purifying selection (Ka/Ks < 1), while *rps3* showed elevated Ka/Ks values in some species, suggesting possible relaxed or positive selection. Phylogenetic analysis based on 57 mitochondrial sequences resolved the relationships among the six *Russula* species.

**Discussion:**

This study enriches the mitogenomic resources of Russulaceae and provides a foundation for future phylogenetic and evolutionary studies of *Russula*.

## 1 Introduction

The genus *Russula* belongs to the phylum Basidiomycota, class Agaricomycetes, order Russulales, and family Russulaceae. It is one of the most widely distributed lineages within Russulaceae (Li G. J. et al., 2021; Cheng et al., 2024) and also one of the most important genera of ectomycorrhizal fungi in global forest ecosystems (Li et al., 2018). Ecologically, species of *Russula* frequently form ectomycorrhizal symbiotic associations with plants from multiple families, including Cyperaceae, Fagaceae, and Pinaceae (Gao and Yang, 2010; Avis et al., 2003). This long-term interaction not only closely coordinates with hosts through nutrient cycle regulation supporting the growth of host plants while indirectly influencing soil carbon cycling and ecosystem stability but also expands the host’s nutrient and water absorption range via extensive underground mycorrhizal networks. Additionally, it mediates resource allocation and signal transmission among plants significantly enhancing the host’s resistance to biotic and abiotic stresses such as drought diseases and heavy metal pollution thus serving as a key functional group for maintaining physiological and ecological processes in forest ecosystems (Wang et al., 2015; Benito and González-Guerrero, 2014; Read and Perez-Moreno, 2003). Beyond ecological value, most *Russula* species possess both edible and medicinal properties (Liu et al., 2024). The polysaccharide components in their fruiting bodies exhibit inhibitory effects against pathogenic bacteria such as *Staphylococcus aureus* and *Escherichia coli*, and also demonstrate hypolipidemic, antitumor, and antiviral activities, with some species showing potential anticancer effects (Li Y. M. et al., 2021). However, it should be noted that not all species of *Russula* are safe for consumption; common toxic species include *R. japonica*, *R*. *subnigricans* Hongo, and *R*. *senecis* (Wu et al., 2019). In summary, *Russula* holds significant value in ecological maintenance, resource utilization, and bioactive compound development, making it a fungal lineage worthy of in-depth research.

Traditional classification within the genus *Russula* has primarily relied on morphological characteristics, such as the size and color of the pileus, the color and thickness of the context, and the presence or absence of color changes upon injury. Among these, the morphological classification systems established by Romagnesi (1987) are widely accepted. However, due to the high morphological diversity and subtle interspecific macroscopic differences within the genus, identification based solely on traditional basidiocarp characteristics poses significant challenges, complicating taxonomic efforts (Carneiro de Melo Moura et al., 2019).

Consequently, advancements in molecular biology have substantially refined the classification methods for *Russula* species. Researchers now commonly employ sequence-based identification. In recent years, the introduction of molecular techniques, such as multi-gene phylogenetic analyses based on the nuclear ribosomal internal transcribed spacer (ITS) region, has provided valuable and complementary data for the identification of *Russula* species (Li et al., 2019). More importantly, the mitochondrial genome (mitogenome) has emerged as a novel molecular marker system. Its unique evolutionary characteristics offer multidimensional advantages for fungal phylogenetic studies (Basse, 2010; Wang et al., 2018) Initially, its genomic architecture exhibits relative stability and conservation, characterized by a moderate evolutionary rate, thereby yielding dependable phylogenetic signals (Burger et al., 2003). Secondly, the mitogenome generally exists in a haploid form, circumventing the complexities of heterozygosity and recombination found in the nuclear genome, thus providing clearer evolutionary pathways for phylogenetic analysis. The mitogenome evolves independently of the nuclear genome, providing unique evolutionary perspectives. It is relevant across many evolutionary timelines, adept at elucidating both recent speciation events and more profound evolutionary history (Bullerwell and Gray, 2004; Havird and Sloan, 2016). Mitogenomes offer considerable potential for biodiversity study and phylogenetic classification (Zhang et al., 2023). This study addresses the deficiency in *Russula* mitogenomic data and elucidates interspecific evolutionary relationships, hence offering significant academic support for studies on this genus. Future research should broaden the spectrum of species examined and investigate more thoroughly the connections between their genetic data and ecological roles.

## 2 Materials and methods

### 2.1 Sample collection and identification

The specimen collection protocol employed in this investigation incorporated line-transect and quadrat survey methodologies, and was executed in compliance with the technical standard HJ 628–2011 (Technical Specification for Biological Genetic Resource Collection). Photographs of the habitat were captured during collection, and comprehensive information (including collection date, location, altitude, habitat, and collector) was documented using a standardized labeling system. The macro-morphological traits of the obtained mature *Russula* basidiocarps were recorded according to conventional morphological taxonomy. The parameters encompassed: pileus diameter and hue; context hue, thickness, and color alteration upon injury; lamellae or pore color and width; the presence or absence of annular morphological features; and stipe length, width, hue, and hollowness.

The specimens were placed in breathable paper bags immediately after collection, transported to the laboratory and air-dried naturally at room temperature within 12 h. All dried specimens were put into zip-lock bags with desiccant and stored in dedicated specimen cabinets for long-term preservation. Detailed collection information of all six specimens, including locality, altitude, longitude and latitude, habitat, collection date and voucher number, is summarized in Supplementary Table S1. All voucher specimens are permanently preserved in the Herbarium of Guizhou Medical University.

### 2.2 Genomic DNA extraction

Genomic DNA was extracted from the *Russula* species using the Solarbio Plant Genomic DNA Extraction Kit, following the manufacturer’s instructions. Briefly, the basidiocarps were first surface-sterilized and ground into a fine powder. An appropriate amount of the sample was then weighed and subjected to lysis and digestion using the provided lysis buffer and proteinase. Subsequently, impurities were removed by passing the lysate through a specific adsorption column. The bound DNA was then thoroughly washed, and the column was dried to eliminate any residual ethanol from the wash buffer, thus preventing potential interference in downstream applications. Finally, high-quality genomic DNA was eluted. The purity and concentration of the extracted DNA were measured using a UV spectrophotometer to ensure they met the requirements for subsequent experiments.

### 2.3 Species identification

The accurate identification of *Russula* species is often challenged by the existence of species complexes. In this study, all specimens were first identified via comprehensive morphological observation following standard fungal taxonomic criteria, including pileus, context, lamellae and stipe characteristics. Morphological identification results were further verified by ITS molecular sequencing.

Species-specific primers for *Russula* were designed using Primer Premier 5 software, and the target samples were subjected to polymerase chain reaction (PCR) amplification. The specific reaction conditions for these *Russula*-specific primers are provided in Supplementary Table S2. Following verification by agarose gel electrophoresis, the PCR amplification products were sent to Tsingke Biotechnology Co., Ltd. for purification. The sequences were subsequently determined using the bidirectional sequencing method. Based on the obtained ITS sequences, phylogenetic trees of the six *Russula* species were constructed (Supplementary Figure S1), which clearly distinguished the tested species from closely related taxa. The newly obtained ITS sequences for the six *Russula* species have been deposited in GenBank under accession numbers PZ205971-PZ205976.

### 2.4 Whole-genome sequencing

The extracted DNA samples were stored in PCR tubes, refrigerated with dry ice, and subsequently shipped to a sequencing service provider (Berry Genomics, Beijing) for whole-genome next-generation sequencing. Following the quality evaluation of the DNA samples, suitable specimens were chosen for subsequent procedures. Each sample was independently constructed into a library and sequenced on the Illumina NovaSeq 6,000 platform in rapid run mode, with an insert size of 300 bp and a paired-end 150 bp (150 PE) sequencing strategy. A minimum of 6 Gb of raw data was generated for each sample, with a Q30 > 83%. The techniques encompassed DNA shearing, library preparation, sequencing, and data analysis. Raw reads were filtered to remove adapter sequences and low-quality reads using Trimmomatic v0.39. Only clean reads were retained for subsequent mitogenome assembly.

### 2.5 Mitogenome assembly and annotation

The mitogenomes were assembled using Geneious Prime R9 2023.2.1 software. *R*. *abietina* (NC_037774) was selected as the reference because it was a complete and well-annotated *Russula* mitogenome available in GenBank at the time of analysis. The Map to Reference function was employed for the primary assembly with a K-mer size of 31 and a minimum coverage threshold of 10×, while the *De Novo* Assemble function was utilized for comparative alignment tasks (Wang et al., 2025a).

For mitogenome annotation, preliminary prediction of transfer RNA (tRNA) genes and the 15core protein-coding genes (PCGs) and ribosomal RNAs (rRNAs) was performed using MITOS (Bernt et al., 2013) and MFannot (Lang et al., 2023). Both tools utilized the Mold/Protozoan mitochondrial genetic code (translation Table 4). tRNA genes were further annotated using tRNAscan-SE with the mitochondrial model and default search parameters (Lowe and Chan, 2016). Subsequently, manual curation of the automated predictions was conducted within Geneious Prime R9 2023.2.1. This process involved open reading frame (ORF) prediction via the Find ORFs function and careful comparison with annotation data from closely related, published species. Finally, physical maps of the six *Russula* mitogenomes were generated using OGDraw v1.3.1 (Lohse et al., 2013).

### 2.6 Comparative analysis of *Russula* mitogenome

The secondary structures of mitochondrial tRNAs were visualized based on the results from tRNAscan-SE. Using MEGA 7.0 software and the Mold/Protozoan genetic code (translation Table 4), we conducted several analyses on the 15 core PCGs from the six species. First, the overall mean genetic distances (K2P), codon usage frequency, and codon usage bias were calculated. Furthermore, nucleotide composition bias was assessed using the formulas: AT-skew = (A − T)/(A + T) and GC-skew = (G − C)/(G + C) (Wang et al., 2017). Additionally, the complete mitogenomes of the six species were subjected to collinearity analysis using Mauve. Finally, the synonymous (Ks) and non-synonymous (Ka) substitution rates for the 15 core PCGs were calculated using DnaSP v6.10.01 (Rozas et al., 2017). The Mold/Protozoan genetic code (translation Table 4) was applied. For each PCG, multiple sequence alignments were manually inspected, and codons containing gaps or ambiguous sites were removed to avoid biased substitution estimates. The Nei-Gojobori method with Jukes-Cantor correction was employed for Ka/Ks calculation. Given the exploratory and descriptive nature of this analysis, no formal hypothesis testing was performed across genes, and thus no multiple testing correction was applied.

### 2.7 Phylogenetic relationship reconstruction and analysis

We first conducted a comparative analysis of the basic mitogenome structure, including assessments of GC content, AT/GC skewness, and gene number and order. Based on different datasets, sequences were aligned using MAFFT 7.0 and subsequently trimmed using trimAl (Katoh et al., 2019). The aligned sequences were concatenated using MEGA 7.0 (Kumar et al., 2016) to construct two datasets: the PCGs dataset and the PCG12 dataset (PCGs with third codon positions removed). Sequence heterogeneity was assessed using AliGROOVE (Kück et al., 2014) with the default BLOSUM62 matrix; scores above 0 indicate low heterogeneity, and no taxa or sites were excluded. Finally, phylogenetic relationship were reconstructed using both the Maximum Likelihood (ML) and Bayesian Inference (BI) methods. BI analysis was performed in MrBayes v3.2.6 (Ronquist et al., 2012) with two independent MCMC runs (10 million generations, sampling every 1,000 trees, 25% burn-in), and ML analysis in IQ-TREE v1.6.3 (Nguyen et al., 2015) with ultrafast bootstrap (1,000 replicates). The resulting trees were visualized and edited using FigTree v1.4.0.

## 3 Results and analysis

### 3.1 Mitogenome composition of six *Russula* species

The documented length of *Russula* mitogenomes varies from 43,430 to 69,432 bp. This study presents the complete mitogenomes of six previously unreported *Russula* species (Supplementary Table S3). Similar to other published species, these six mitogenomes contained a conserved set of 15 core PCGs. These included the cytochrome b gene (*cob*), three ATP synthase subunit genes (*atp6*, *atp8*, *atp9*), three cytochrome c oxidase subunit genes (*cox1*-*cox3*), seven NADH dehydrogenase subunit genes (*nad1*-*nad6* and *nad4L*), and one ribosomal protein gene (*rps3*). Additionally, two rRNA genes—the small subunit rRNA (*rrnS*) and large subunit rRNA (*rrnL*)—were identified. All these genes were located on the major strand.

**Figure 1:**
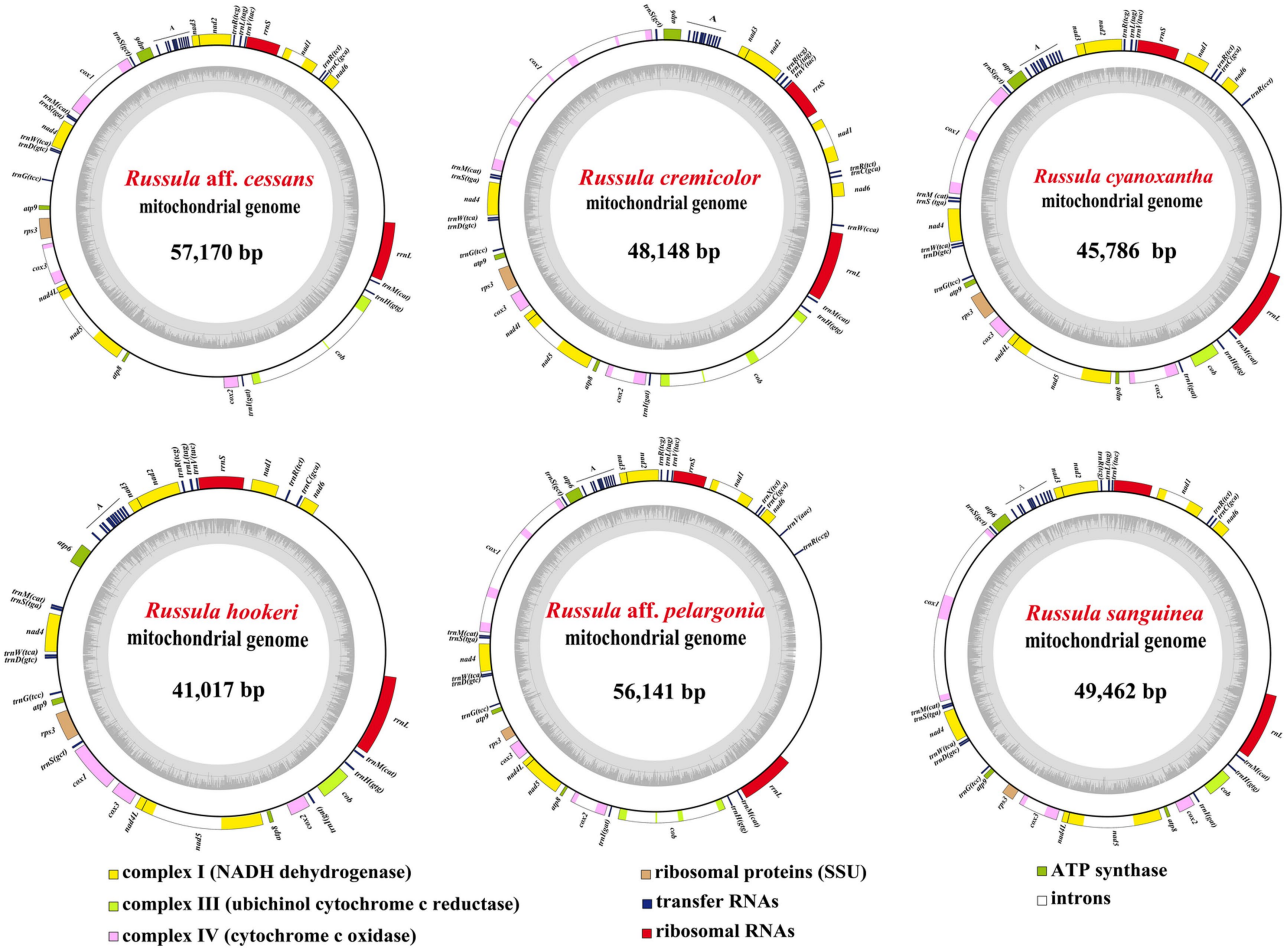
In the circular maps of the six mitochondrial genomes. Genes are denoted by distinct colored blocks. The blocks depicted outside the circles represent genes located on the forward strand. The specific block labeled A (from left to right) corresponds to the following tRNA genes: *trnE*, *trnM*, *trnL*, *trnN*, *trnP*, *trnY*, *trnK*, *trnQ*, *trnT, trnF,* and *trnA*.

Among the six *Russula* species assembled in this study, the previously unsequenced species *R. hookeri* possessed the smallest mitogenome at 41,017 bp, while *R*. aff. *Cessans* had the largest, reaching 57,170 bp (Figure 1). This size variation indicates significant changes in mitogenome length during the evolution of *Russula* fungi. The mitogenomes of *Russula* species exhibited a relatively relaxed structure. With the exception of *R*. *cremicolor*, all species contained only a single nucleotide overlap between the *nad2* and *nad3* genes, and another between the *nad4L* and *nad5* genes.

### 3.2 Analysis of tRNA secondary structures

**Figure 2:**
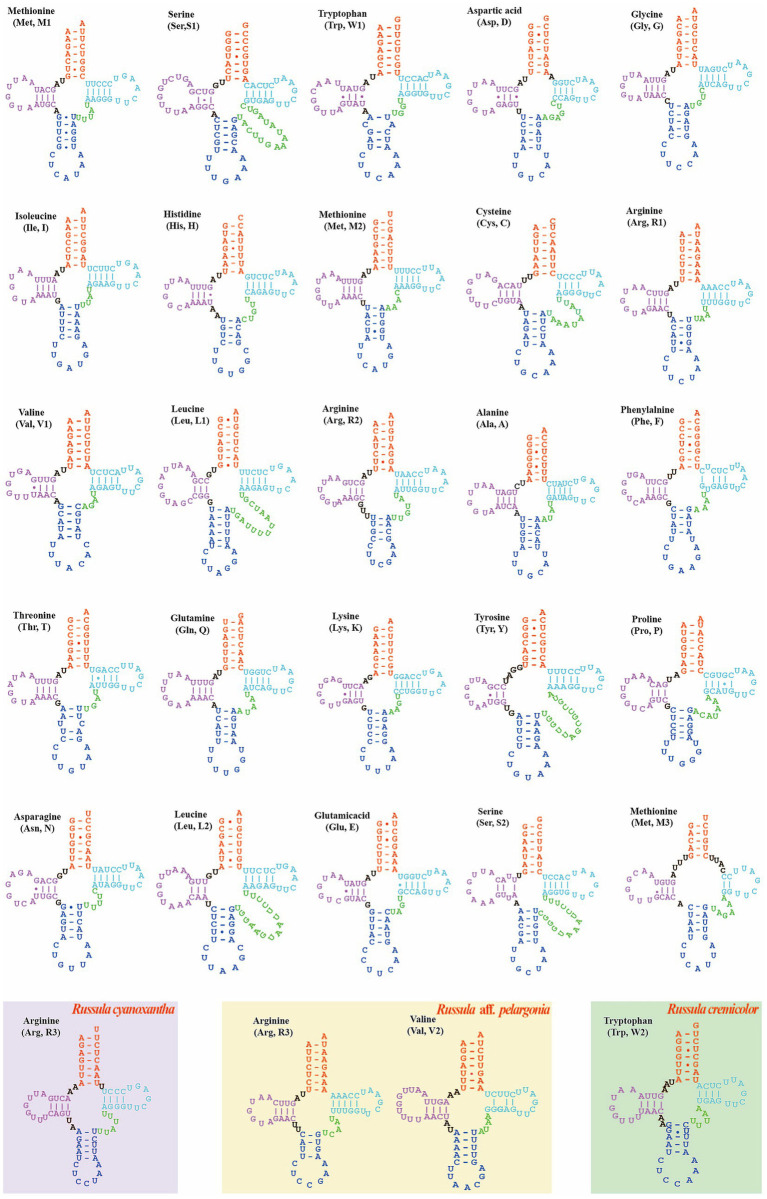
Secondary structures of tRNAs from the six *Russula* species. A core set of 25 tRNA genes with strictly conserved anticodons was shared across the six *Russula* species, as exemplified by *R*. aff. *Cessans*. Sequence polymorphisms among these tRNAs were primarily confined to nucleotide variations in the loop regions (see Supplementary material). The accompanying figure annotates species-specific tRNA types in distinct colors.

The number of transfer RNAs (tRNAs) in the mitogenomes of the six *Russula* species ranged from 25 to 27 (Figure 2; Supplementary Figures S2–S7). Their secondary structures were visualized and all exhibited the highly conserved cloverleaf configuration. The length of individual tRNAs varied from 69 to 86 bp, with the combined total length of all tRNAs ranging from 1,707 to 1,945 bp, accounting for 3.22 to 4.53% of the total mitogenome length.

Analysis revealed that 25 tRNA genes were shared among all six species. Among these, methionine (*Met*) was encoded by three tRNA genes bearing the identical anticodon CAU. Leucine (*Leu*; with anticodons UAG and UAA), arginine (*Arg*; UCU and UCG), and serine (*Ser*; UGA and GCU) were each encoded by two distinct tRNA genes with different anticodons. Additionally, species-specific variants were noted: *R*. *cremicolor* included one extra tRNA*^Trp^* (CCA), *R*. *cyanoxantha* had one extra tRNA*^Arg^* (CCU), and *R*. aff. *pelargonia* exhibited two additional tRNAs, specifically tRNA*^Arg^* (CCG) and tRNA*^Val^* (AAC).

### 3.3 Analysis of codon usage frequency

**Figure 3:**
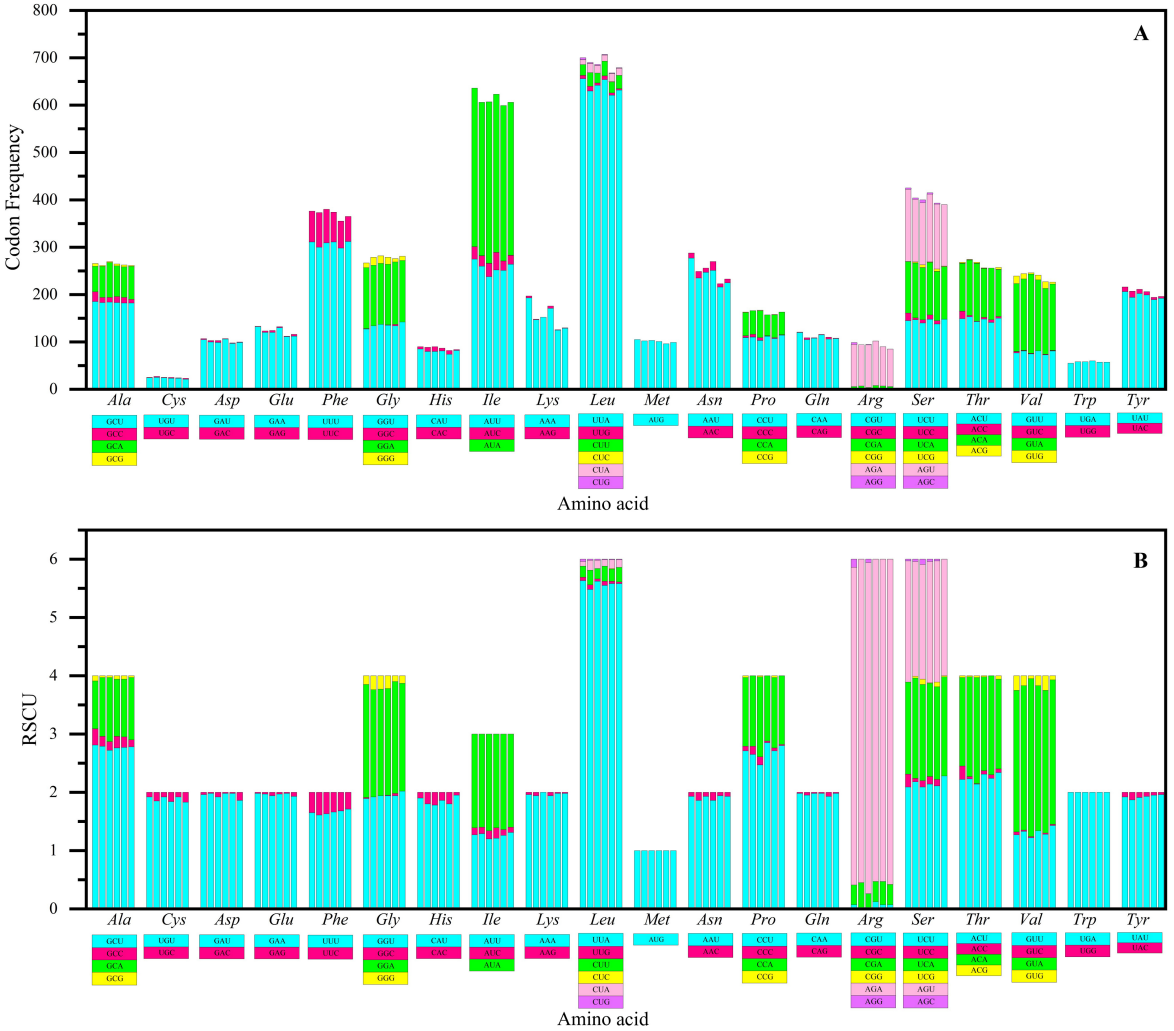
Codon usage characteristics of the six *Russula* species. **(A)** The codon usage frequency of each amino acid; **(B)** The relative synonymous codon usage (RSCU) values. From left to right: *R*. *cremicolor*, *R. sanguinea*, *R*. aff. *pelargonia*, *R*. *cyanoxantha*, *R*. aff. *Cessans*, *R. hookeri*. Different colored boxes represent different codons.

The amino acid usage frequency (Figure 3A) indicated that *Leu* was the most frequently used, followed by *Ile*, while *Cys* was the least utilized. Preference analysis (Figure 3B) revealed that all six species shared 27 high-frequency codons (RSCU > 1.00). The most frequently used codons were UUA (*Leu*) and AGA (*Arg*), which represents a notable characteristic of *Russula* mitogenomes. Analysis showed that codons ending with U or A were generally used more frequently, while those ending with G or C exhibited lower usage frequencies (Wu et al., 2020; Wang et al., 2025b). 15 core PCGs consistently used ATG as the initiation codon across all six species. For termination codons, TAA was used in the vast majority of PCGs. only three sequences—*atp9* of *R*. *cyanoxantha*, and *nad4* and *cob* of *R*. *cremicolor*—used TAG instead of TAA. This indicates that TAA is highly conserved as the termination codon in *Russula* mitogenomes.

### 3.4 Analysis of mitogenome composition

**Figure 4:**
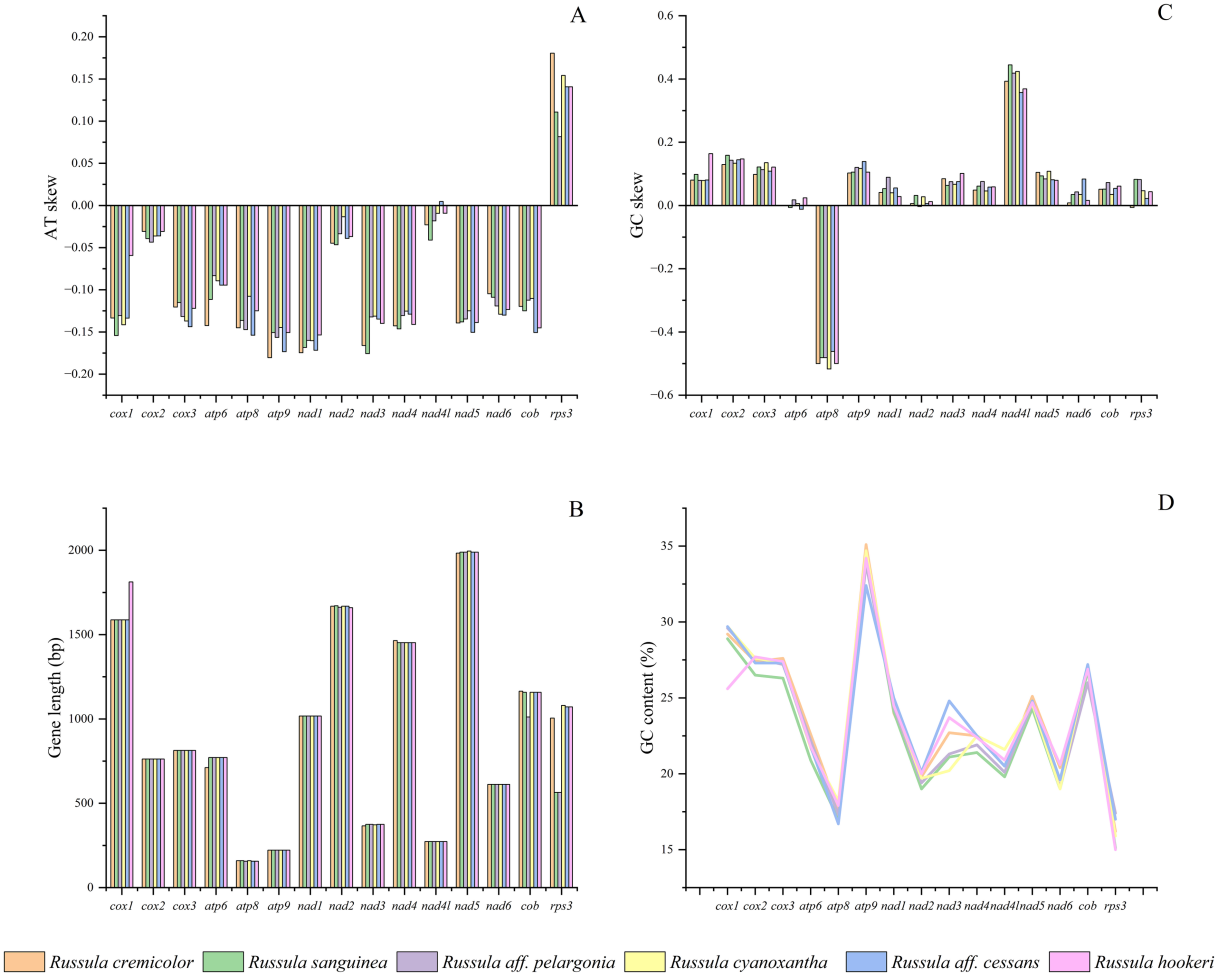
Comparative analysis of mitochondrial genome PCGs from six *Russula* species. **(A)** AT-skew; **(B)** GC-skew; **(C)** PCG length variation; **(D)** GC content of PCGs.

A total of 10 complete *Russula* mitogenomes were retrieved from NCBI, together with the 6 newly sequenced species from this study. Among the NCBI sequences, one (*Russula sanguinea*, PP933239) was excluded from the selective pressure and collinearity analyses because it represented the same species as one of our newly sequenced samples. Regarding nucleotide skewness, AT-skew values were negative in 13 core PCGs, consistently positive in *rps3*, and variable in *nad4L* (predominantly positive with only one instance being negative) (Figure 4A). GC-skew values were positive in 11 core PCGs, consistently negative in *atp8*, and variable (mostly positive, occasionally negative) in *atp6*, *nad2*, and *rps3* (Figure 4B). Among the 15 core PCGs in the six *Russula* species, length variations were detected in eight genes (Figure 4C). The *rps3* gene exhibited the greatest length variation among the 15 core PCGs, followed by the *cox1* gene. The GC content of the 15 core PCGs varied across the six *Russula* species (Figure 4D), indicating mutations have occurred in the core PCGs of the *Russula* mitogenomes. Among the 15 core PCGs examined, *atp9* possessed the highest GC content (average 34.07%), whereas *rps3* had the lowest (average 16.1%).

### 3.5 Gene arrangement and collinearity analysis

**Figure 5:**
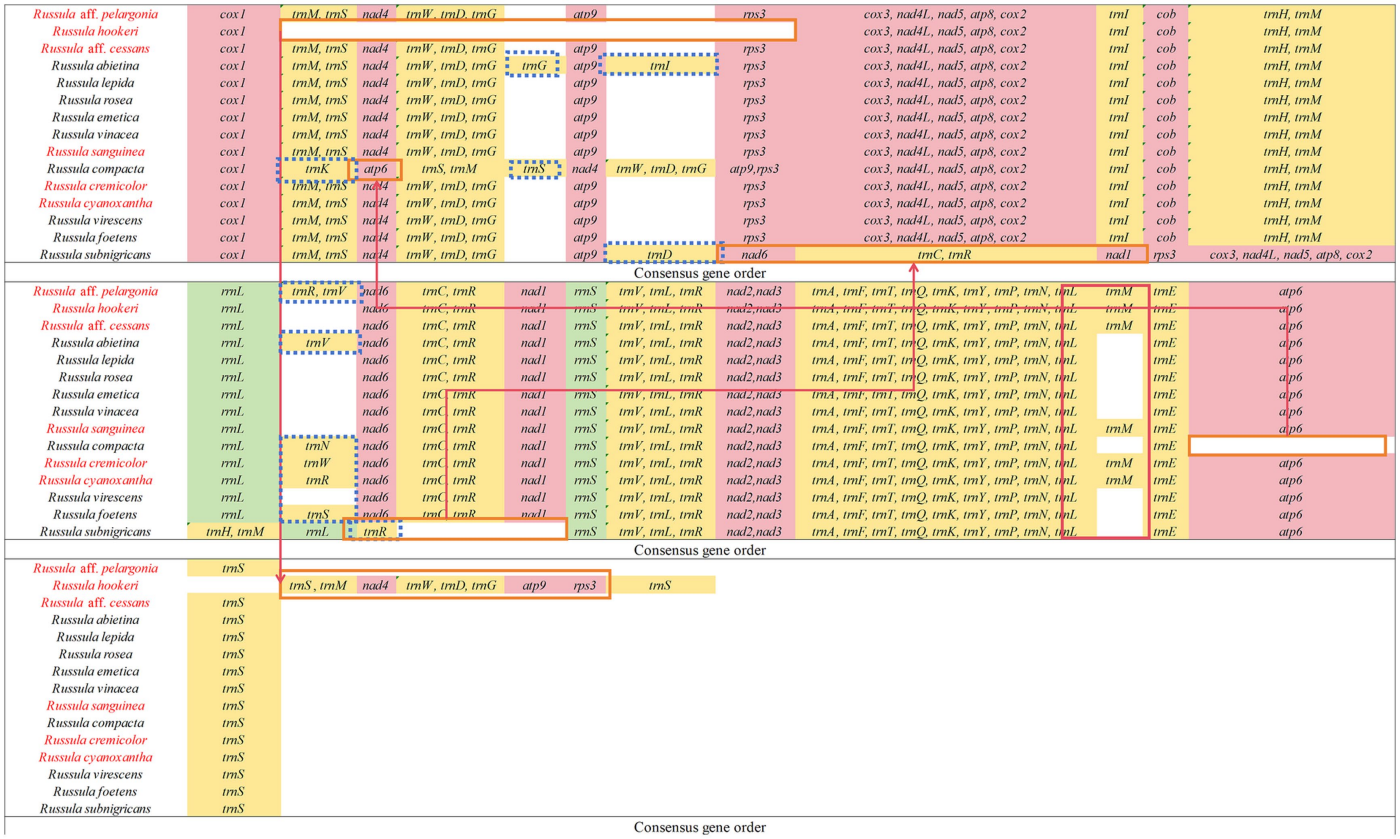
Gene arrangement order of the 15 *Russula* species. The blue dashed boxes denote inserted genes, while the orange boxes indicate displaced genes. The genes corresponding to the red boxes are shared at the same locus among the six species included in this study; these genes are present in the genomes of other species but have not been annotated.

Based on phylogenetic relationships, gene order analysis was performed on 15 core PCGs, 2 rRNAs, and approximately 25 tRNAs from nine *Russula* mitogenomes retrieved from the NCBI database and six *Russula* genomes sequenced in this study. The results showed that three species (*R*. aff. *pelargonia*, *R. hookeri*, *R. sanguinea*) and four species (*R. lepida*, *R. rosea*, *R*. *emetica*, *R. vinacea*) exhibited identical gene orders, with the former group containing only one additional *trnM* gene compared to the latter; slight differences in gene order were observed among the remaining species (Figure 5). It was observed that the relative positions of 23 tRNA genes in these mitogenomes were highly conserved, and the order of the 15 core PCGs was essentially as follows: *cox1* - *nad4* - *atp9* - *rps3* - *cox3* - *nad4l* - *nad5* - *atp8* - *cox2* - *cob* - *nad6* - *nad1* - *nad2* - *nad3* - *atp6*. Additionally, some irregularly distributed tRNA genes were randomly scattered in the mitogenomes of several species. Three major gene rearrangements involving positional shifts were identified in the gene order: first, in *R. hookeri*, a cluster of genes (including *trnS*, *trnM*, *nad4*, *trnW*, *trnD*, *trnG*, *atp9*, and *rps3*) shifted from downstream of *cox1* to downstream of atp6; second, in *R*. *subnigricans*, genes *nad6, trnC*, *trnR*, and *nad1* shifted from downstream of *rrnL* to upstream of *rps3*; third, in *R. compacta*, the *atp6* gene shifted from downstream of *trnE* to upstream of *trnS* and *trnM*.

**Figure 6:**
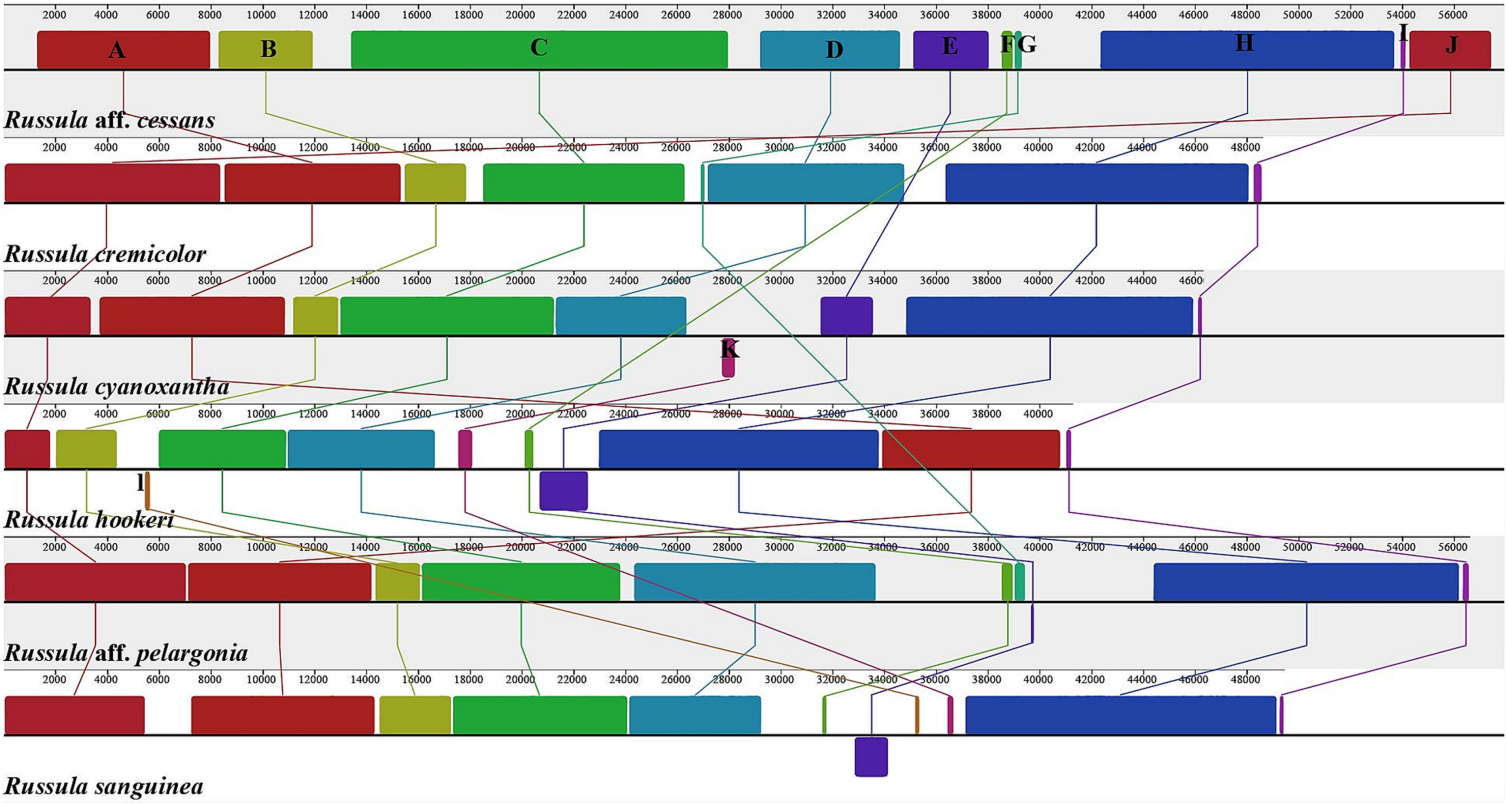
Collinearity analysis of the six *Russula* species. Different colored blocks represent distinct homologous regions, and the size of each block corresponds to the size of the homologous region.

Comparative analysis of the mitogenomes of six *Russula* species identified a total of 12 homologous regions (Figure 6). The results showed that most homologous regions exhibited high overall conservation, with the positions of core blocks being relatively fixed. However, there were significant differences in the size of each region, showing no uniformity. Only a few homologous regions were species-specific; for instance, two homologous blocks labeled K and I were present exclusively in the mitogenomes of a small number of *Russula* species. Specifically, the color block I was randomly distributed in *R. hookeri* and *R. sanguinea*, while the color block K was present in the mitogenomes of *R. hookeri*, *R. sanguinea*, and *R*. *cyanoxantha*.

### 3.6 Analysis of selective pressure and evolutionary rates

**Figure 7:**
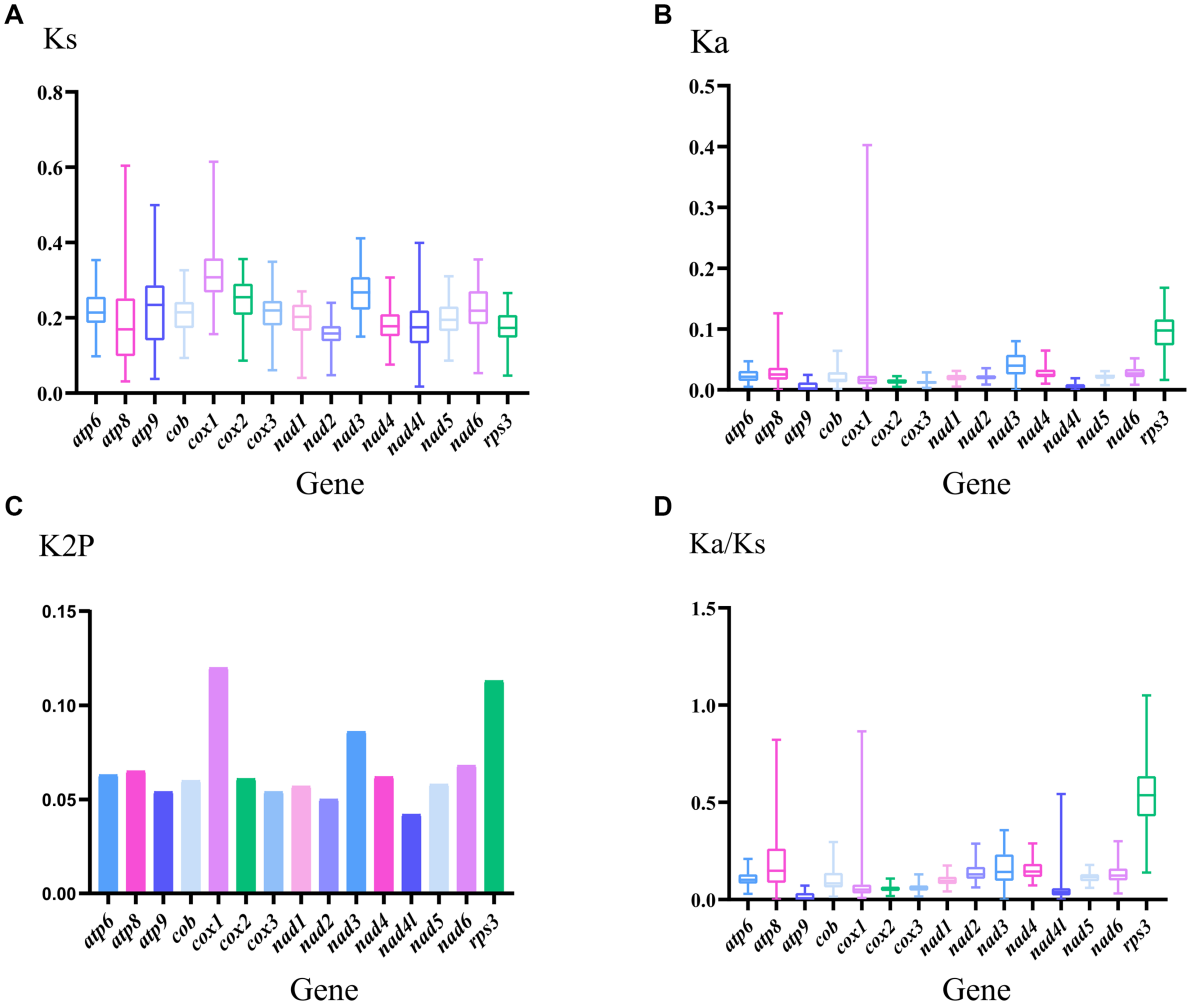
Analyses of evolutionary rates and genetic distances of 15 core PCGs across 15 *Russula* species. **(A)** Ks values of each PCG; **(B)** Ka values of each PCG; **(C)** Ka/Ks ratios of each PCG; **(D)** interspecific K2P genetic distances of each PCG.

Evolutionary rate and genetic distance analyses were performed on the 15 PCGs of the aforementioned 15 *Russula* species. The results are as follows: Among all PCGs, the *cox1* gene exhibited the highest synonymous substitution rate (Ks) (Figure 7A), and its nonsynonymous substitution rate (Ka) was also significantly higher than that of other PCGs (Figure 7B). The *nad4L* gene showed the smallest genetic distance, while the *atp9* gene had the lowest Ka/Ks ratio, indicating that these two genes are highly conserved during evolution. Except for the *rps3* gene, the Ka/Ks ratios of the remaining 14 PCGs were all less than 1, suggesting that these genes are mainly constrained by purifying selection in the evolutionary process (Figure 7C). Furthermore, the K2P genetic distances of the *cox1* and *rps3* genes were significantly higher than those of other PCGs (Figure 7D).

### 3.7 Phylogenetic heterogeneity analysis

**Figure 8:**
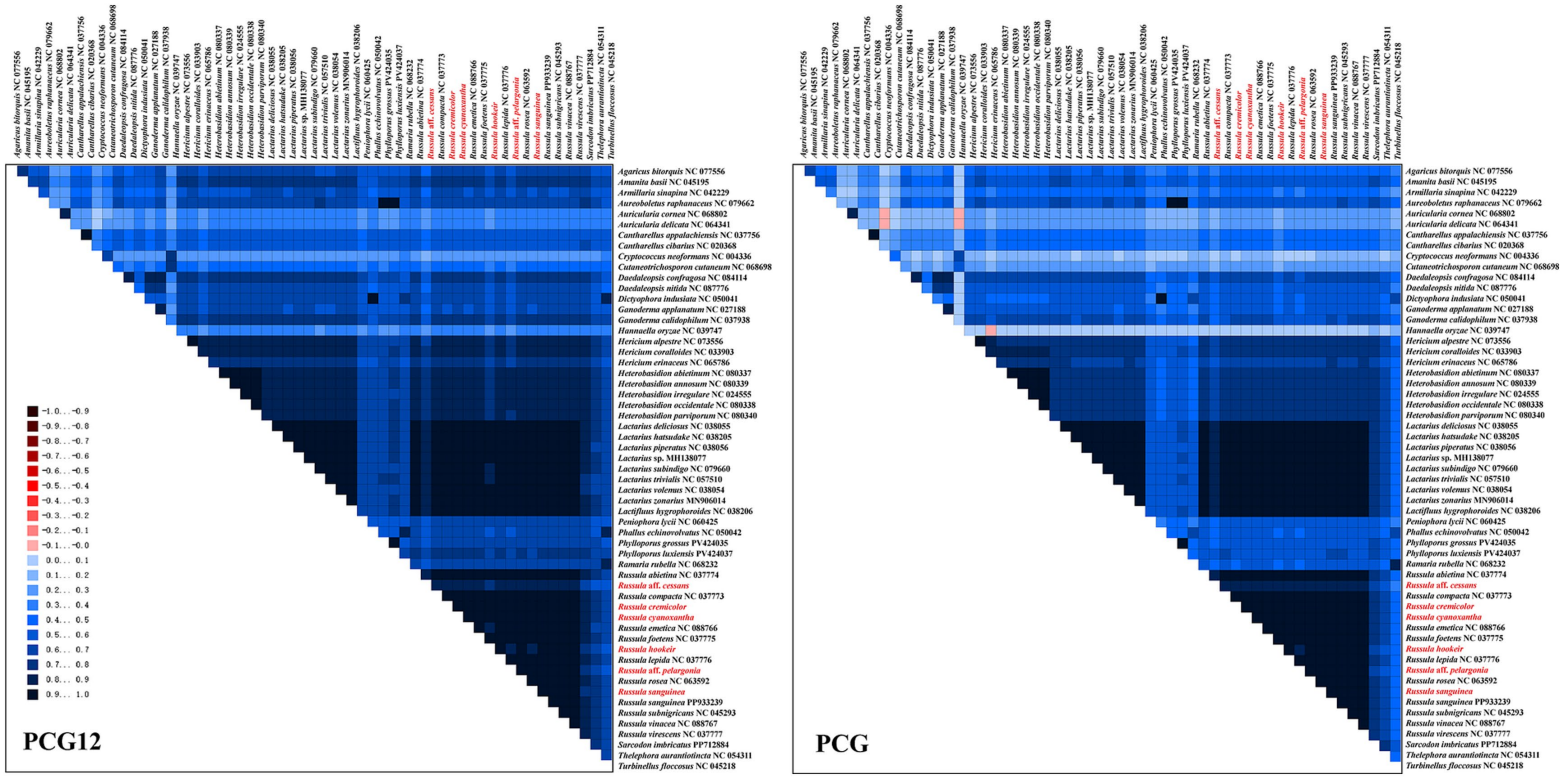
Heterogeneity analysis of the PCG12 and PCG datasets for phylogenetic relationship reconstruction. Deep red to deep blue indicates a change in heterogeneity, corresponding to a shift from heavy to light values.

Prior to phylogenetic reconstruction, sequence compositional heterogeneity analysis was conducted on the two concatenated mitogenome datasets (PCG12 and PCG) of 57 included taxa (Figure 8). The results revealed that the majority of species pairs exhibited high sequence homology and low compositional heterogeneity in both datasets, with only a small number of interspecific pairs showing minor sequence divergence. All 57 mitogenome sequences contain the complete set of 15 core PCGs, with no missing genes in the concatenated alignment. The AliGROOVE analysis used the default BLOSUM62 scoring matrix; heterogeneity scores above 0 indicate low heterogeneity. Because the overall heterogeneity was low, no taxa or sites were excluded. This confirmed that the two datasets contained stable evolutionary signals and were suitable for subsequent phylogenetic inference.

### 3.8 Phylogenetic analysis

**Figure 9:**
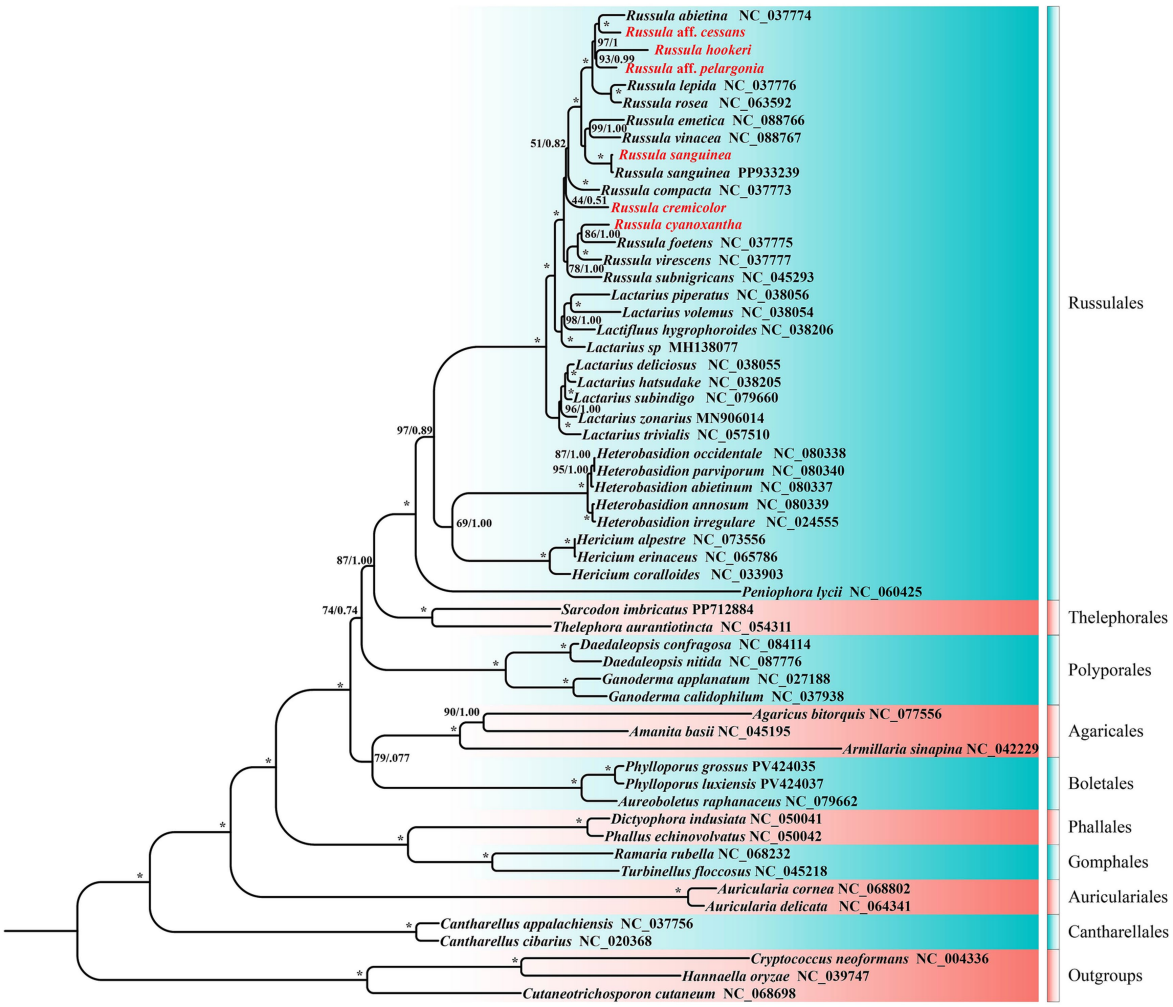
Phylogenetic relationship reconstruction of Agaricomycetes inferred from the mitochondrial PCG datasets using ML methods. Clades containing species sequenced in this study are highlighted in red. Nodal support values are shown as Maximum Likelihood bootstrap values.

Four phylogenetic trees were constructed in this study based on different datasets and reconstruction methods. Only the topology with optimal performance is displayed in the main text (Figure 9), the remaining trees are provided in Supplementary Figures S8–S10. To ensure phylogenetic robustness, we selected Tremellomycetes as the outgroup based on multigene phylogenies placing it as the sister lineage to Agaricomycetes (Hibbett, 2006). To mitigate potential long-branch attraction, we (i) used two datasets (PCG and PCG12, the latter with third codon positions removed), (ii) applied a site-heterogeneous model (LG + F + R) in IQ-TREE, and (iii) visually inspected trees for artifacts. The phylogenetic topologies reconstructed via Bayesian Inference (BI) and Maximum Likelihood (ML) were highly congruent, and the majority of deep nodes were strongly supported (BI posterior probability = 1, ML bootstrap>86), indicating that the overall phylogenetic framework is robust. All included taxa of Agaricomycetes were resolved into well-supported monophyletic clades, which were consistent with their respective taxonomic orders. The Tremellomycetes taxa formed a fully supported monophyletic clade, which was stably recovered as the outgroup of the ingroup Agaricomycetes. The order Russulales was recovered as a robust, fully supported monophyletic clade, which was further divided into four well-supported family-level monophyletic lineages: Russulaceae, Bondarzewiaceae, Hericiaceae, and Peniophoraceae. Within the family Russulaceae, three highly supported monophyletic generic clades were clearly delineated: *Russula*, *Lactarius*, and *Lactifluus*. The topological structure recovered in this study was highly congruent with the well-established taxonomic and phylogenetic framework of Russulaceae inferred from nuclear multi-locus data (ITS, LSU and *rpb2*) in previous studies (De Crop et al., 2017). All six newly sequenced *Russula* species from this study were nested within the *Russula* generic clade, forming a stable subclade with other published *Russula* mitogenome sequences.

## 4 Discussion

### 4.1 Characteristics of the mitogenomes in *Russula* fungi

In comparative analyses of mitogenomes among the six *Russula* species examined in this study, we found that the intergenic spacer region between *rrnL* and *nad6* in *R*. *cremicolor* is significantly shorter (1,155 bp) than that in other five species (7,398–13,412 bp). This observation is consistent with the evolutionary paradigm that “Genome reduction is typical in host-restricted symbionts and pathogens” (Moran and Bennett, 2014). A consistent pattern has also been documented in the genus *Amanita*: compared with saprotrophic relatives, ectomycorrhizal *Amanita* species exhibit a lower proportion of intergenic spacers and more compact mitogenomes, a feature regarded as an adaptive trait associated with a symbiotic lifestyle (Li et al., 2020a). Notably, *trnM* was consistently annotated in the *trnE*-*trnL* intergenic spacer across all six *Russula* species, and its conservation within the genus suggests that this region warrants further investigation.

### 4.2 Comparative analysis of mitogenomes in *Russula*

The GC content directly affects the mutation and recombination frequencies of genomes, and it is an important parameter for species evolution (Teng et al., 2023). The GC contents of the 15 core PCGs varied across the mitogenomes of the six *Russula* species, indicating that frequent mutations have occurred in the PCGs of *Russula* mitogenomes (Huang et al., 2024). The observed size variation among *Russula* mitogenomes (41,017 to 57,170 bp) appears to be influenced by intron content variation. This pattern is consistent with the general view that fungal mitogenomes are shaped by both intergenic spacer expansion/contraction and intron dynamics (Li et al., 2023; Sandor et al., 2018). In basidiomycetes specifically, introns have been identified as a major determinant of genome size variation (Himmelstrand et al., 2014).

### 4.3 Evolutionary dynamics and functional constraints of core PCGs

Some additional tRNA genes are randomly distributed in the mitogenomes of certain *Russula* species, and gene displacement has occurred, leading to frequent changes in the evolutionary process of gene rearrangement in *Russula* species (Li et al., 2020b). Notably, elevated Ka/Ks of *rps3* under positive selection has also been reported in the ectomycorrhizal genus *Pisolithus* (Wu et al., 2021). Whether the elevated Ka/Ks observed in *Russula* is similarly linked to host specificity or environmental adaptation remains an open question requiring further investigation. These findings provide important references for phylogenetic analysis and functional gene research of *Russula* species.

### 4.4 Phylogenetic analysis of Russulales based on mitogenomes

Mitogenomes are generally thought to have low rates of genetic recombination, but recombination events have been documented in various eukaryotic lineages, including fungi (Barr et al., 2005). Nevertheless, sequence variations in mitogenomes largely arise from the gradual accumulation of mutations, which endows them with high fidelity of evolutionary signals among species and taxa (Ma et al., 2025).

*Ecological characteristics of Russulaceae lineages*: The three well-supported monophyletic generic clades within Russulaceae recovered in this study (*Russula*, *Lactarius*, and *Lactifluus*) represent well-documented typical ectomycorrhizal fungal lineages. Previous studies have confirmed that species of these three genera can form stable ectomycorrhizal symbioses with a broad range of host plants from multiple families, including Fagaceae, Dipterocarpaceae, and Pinaceae (Wang et al., 2015; Wei et al., 2024; De Crop et al., 2021). All *Russula* taxa recovered in our analysis were nested within the monophyletic genus clade, which is consistent with the well-established ecological and taxonomic framework of this genus (Blom et al., 2009; Liu et al., 2024).

*Phylogenetic implications of the mitogenome dataset*: The results of this study provide new, robust mitochondrial phylogenomic evidence for the evolutionary relationships among the three core genera of Russulaceae: *Russula*, *Lactarius*, and *Lactifluus*. Our findings supplement valuable mitochondrial data for resolving the deep backbone phylogenetic relationships among Russulales lineages, although support at some nodes remains to be improved, indicating that current mitogenomic data are still insufficient to fully resolve intrageneric relationships. This limitation echoes similar challenges observed in other ectomycorrhizal fungal lineages, where certain intrageneric nodes also exhibited only moderate support despite robust family-level topologies. Our phylogenetic framework established herein provides a reliable molecular basis for the taxonomic identification of ectomycorrhizal Russulaceae species, and lays a foundation for the taxonomic revision and comparative mitogenomic analysis of *Russula*. Similar challenges in resolving certain intrageneric nodes have also been observed in other ectomycorrhizal lineages, including *Paxillus* (Li et al., 2020b), where certain internal nodes exhibited only moderate support despite robust family-level topologies. Given the ecological and economic importance of Russulaceae as ectomycorrhizal symbionts and edible fungi (Wang et al., 2015; Li G. J. et al., 2021), future studies should combine expanded taxon sampling with integrative analyses of mitochondrial and nuclear genomic data to clarify persistent taxonomic uncertainties within *Russula* and its allies, and to obtain more comprehensive mitogenomic information.

## 5 Conclusion

This study presents the first assembly and annotation of the complete mitogenomes of six *Russula* species, contributing new foundational data to the mitochondrial genomics research of this genus. Analysis revealed that the mitogenomes of the studied *Russula* species exhibit conserved characteristics in codon usage bias and tRNA secondary structure, with most PCGs under purifying selection. Future research should focus on sequencing additional mitogenomes from phylogenetically informative *Russula* species to resolve the low-support node within the *Russula* clade, and integrating mitogenomic data with existing nuclear markers (ITS, LSU, and *rpb2*) to clarify taxonomic uncertainties within *Russula* and its allies. More mitogenome data will provide strong evidence for species with unresolved phylogenetic relationships, offering further insights into the diversity of *Russula* and related ectomycorrhizal fungi at the genomic level.

## Data Availability

The six newly sequenced mitogenomes were deposited at GenBank under the following accession numbers: *Russula* aff. *cessans* (PX832404); *Russula cremicolor* (PX858194); *Russula cyanoxantha* (PX858195); *Russula hookeri* (PX858196); *Russula* aff. *pelargonia* (PX858197); Russula sanguinea (PX858198).

## Supplementary material

SUPPLEMENTARY FIGURE S1 Phylogenetic relationship reconstruction of the six *Russula* species inferred from the nuclear ribosomal ITS (Internal Transcribed Spacer) sequences using ML method. Sequences of the six *Russula* species newly sequenced in this study are highlighted in red. Nodal support values are shown as Maximum Likelihood bootstrap values.

SUPPLEMENTARY FIGURE S2 *R*. aff. *cessans* tRNA Secondary Structures.

SUPPLEMENTARY FIGURE S3 *R*. *cremicolor* tRNA Secondary Structures.

SUPPLEMENTARY FIGURE S4 *R*. *cyanoxantha* tRNA Secondary Structures.

SUPPLEMENTARY FIGURE S5 *R*. *hookeri* tRNA Secondary Structures.

SUPPLEMENTARY FIGURE S6 *R*. aff. *pelargonia* tRNA Secondary Structures.

SUPPLEMENTARY FIGURE S7 *R*. *sanguinea* tRNA Secondary Structures.

SUPPLEMENTARY FIGURE S8 Phylogenetic tree of Agaricomycetes inferred from the mitochondrial PCG dataset using BI. Newly sequenced *Russula* species are highlighted in red; nodal support values are shown as Bayesian posterior probabilities.

SUPPLEMENTARY FIGURE S9 Phylogenetic tree of Agaricomycetes inferred from the mitochondrial PCG12 dataset using ML. Newly sequenced *Russula* species are highlighted in red; nodal support values are shown as ML bootstrap values.

SUPPLEMENTARY FIGURE S10 Phylogenetic tree of Agaricomycetes inferred from the mitochondrial PCG12 dataset using BI. Newly sequenced *Russula* species are highlighted in red; nodal support values are shown as Bayesian posterior probabilities.
