# Supplementary figures and images for: Complete mitochondrial genomics reveals phylogenetic relationships and mitogenomic features in six ectomycorrhizal Russula species
Source: Front Microbiol. 2026 Jul 10;17:1865163. doi: 10.3389/fmicb.2026.1865163 (PMC13395875; doi:10.3389/fmicb.2026.1865163)

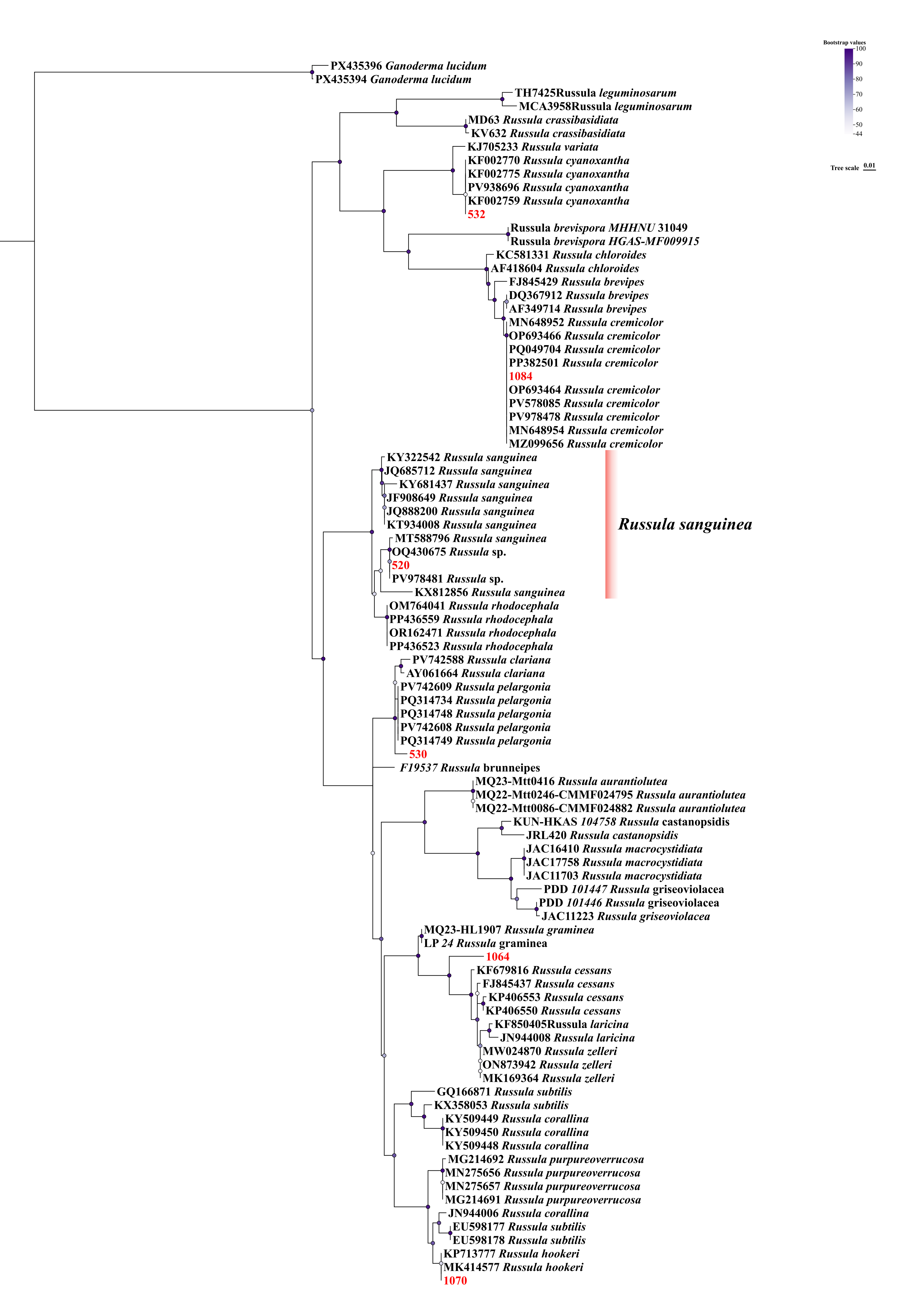

Supplement: SUPPLEMENTARY FIGURE S1 — Phylogenetic relationship reconstruction of the six Russula species inferred from the nuclear ribosomal ITS (Internal Transcribed Spacer) sequences using ML method. Sequences of the six Russula species newly sequenced in this study are highlighted in red. Nodal support values are shown as Maximum Likelihood bootstrap values. [file Image_1.PNG]

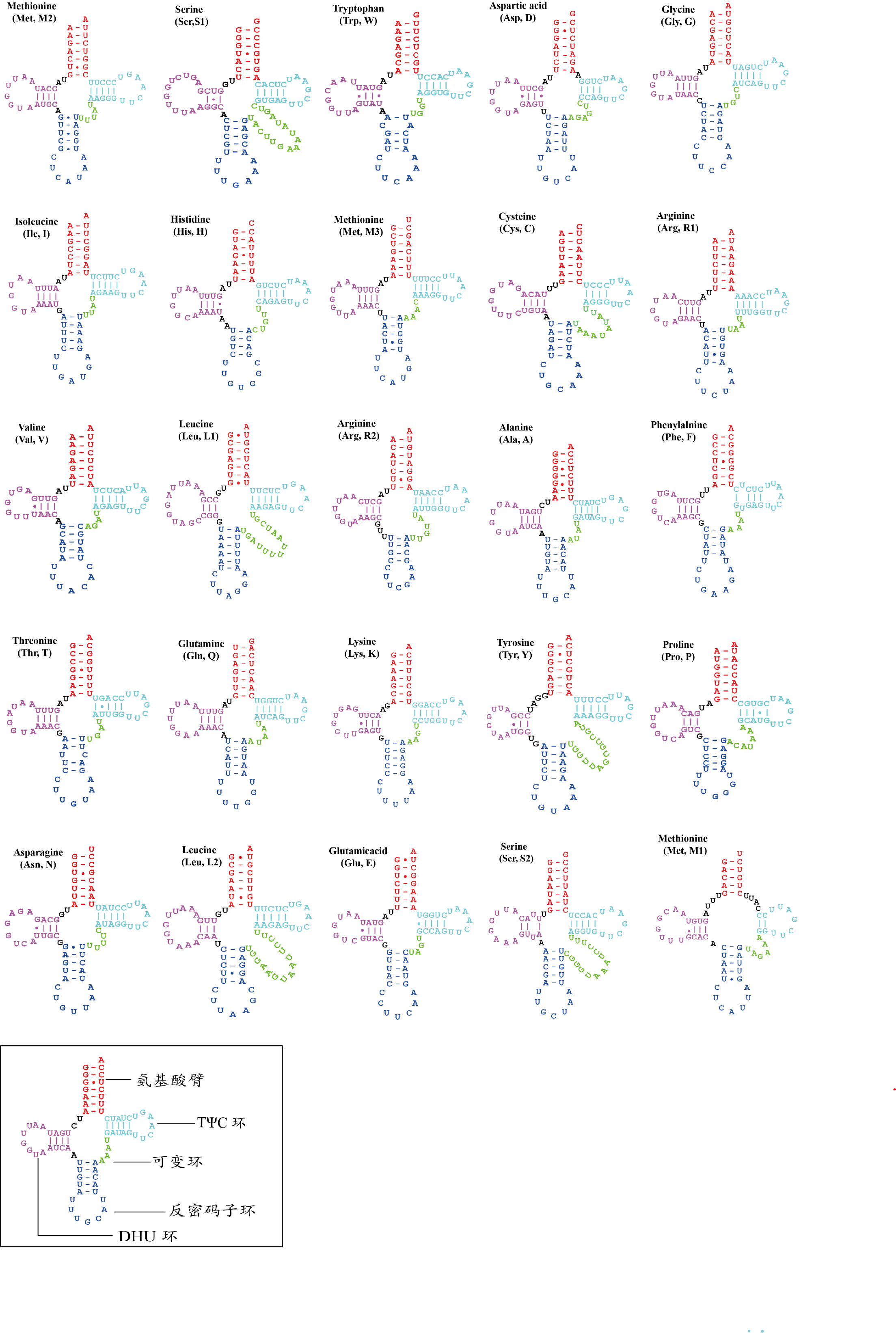

Supplement: SUPPLEMENTARY FIGURE S2 — R. aff. cessans tRNA Secondary Structures. [file Image_2.PNG]

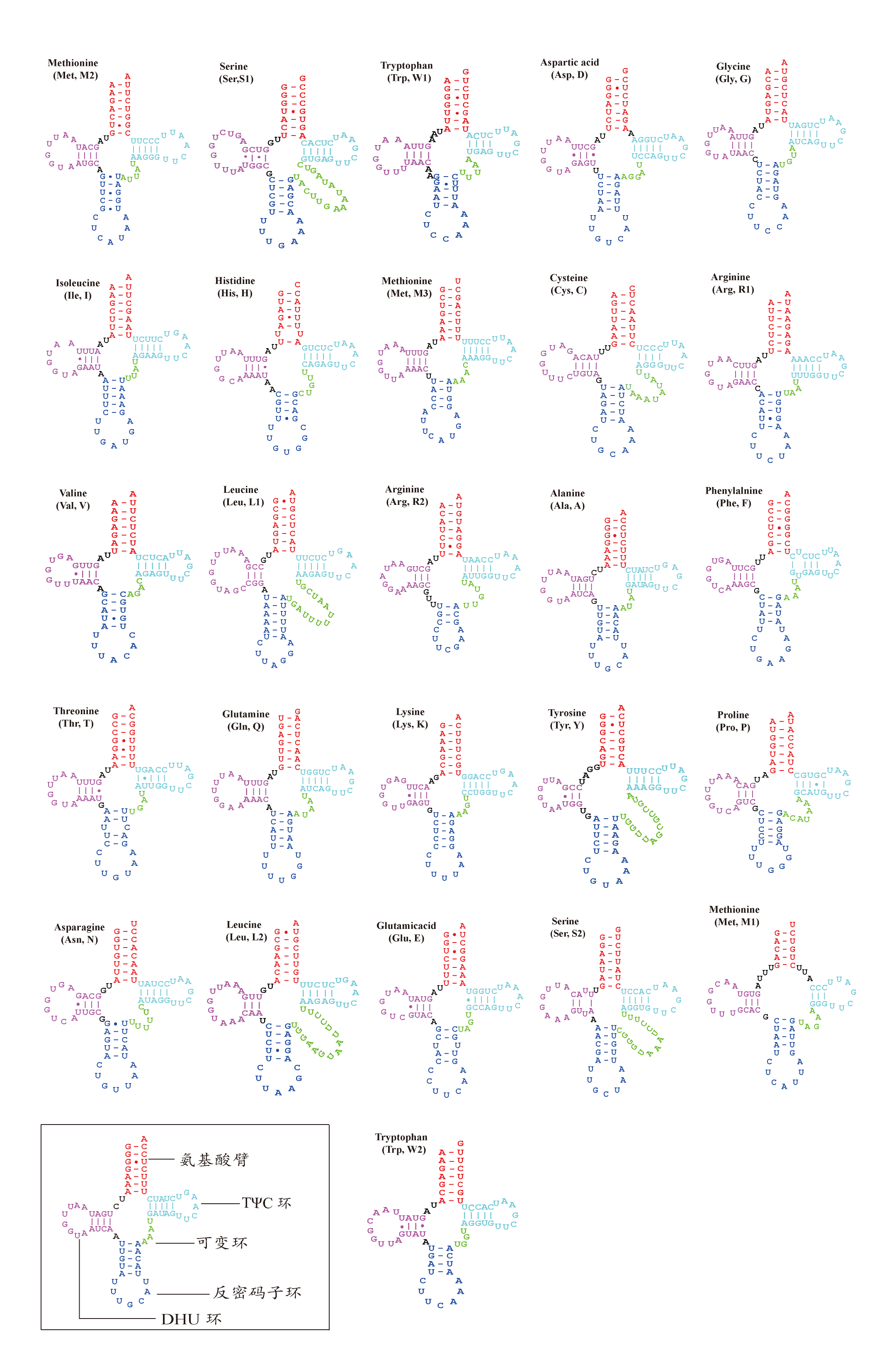

Supplement: SUPPLEMENTARY FIGURE S3 — R. cremicolor tRNA Secondary Structures. [file Image_3.PNG]

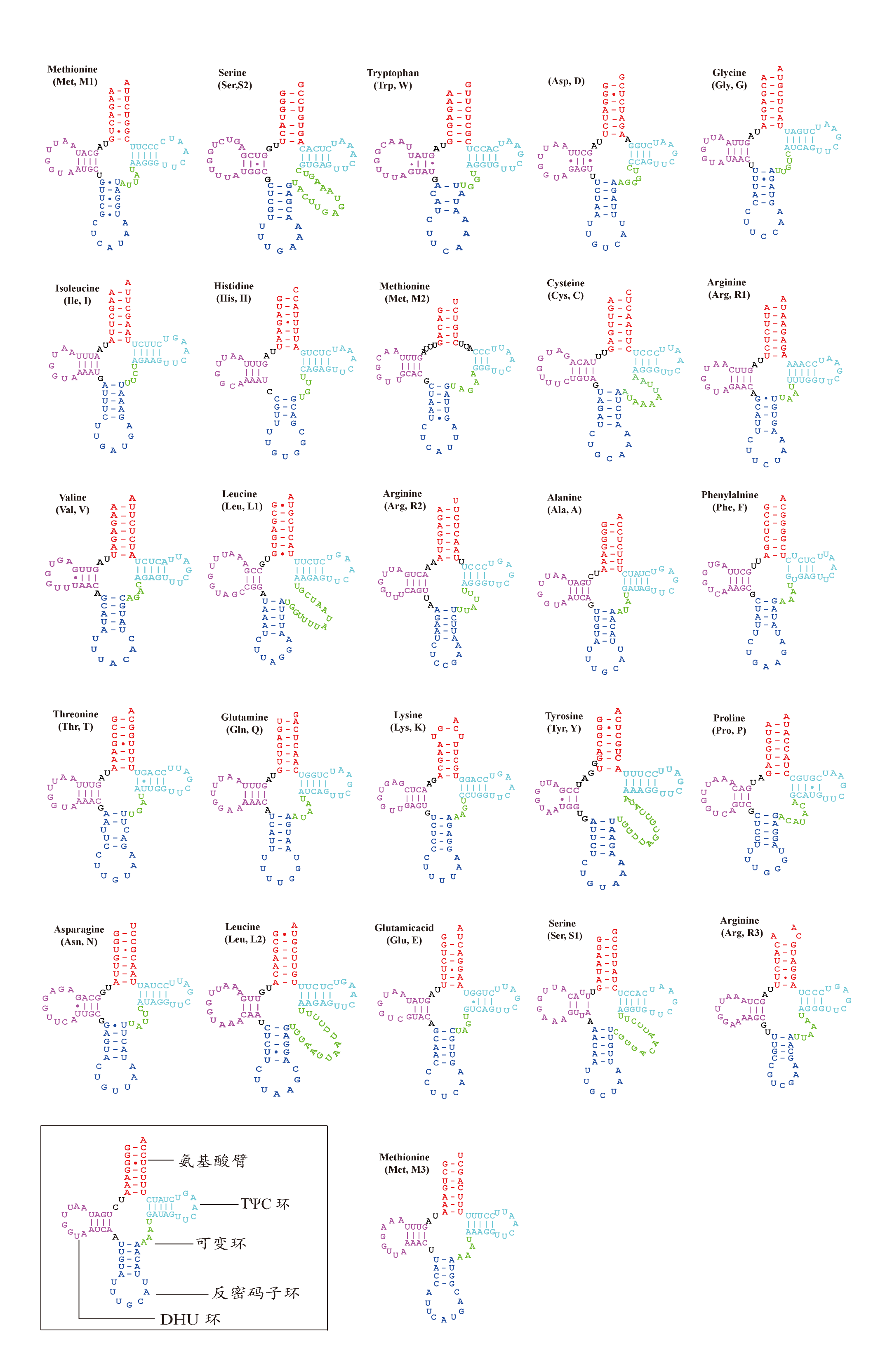

Supplement: SUPPLEMENTARY FIGURE S4 — R. cyanoxantha tRNA Secondary Structures. [file Image_4.PNG]

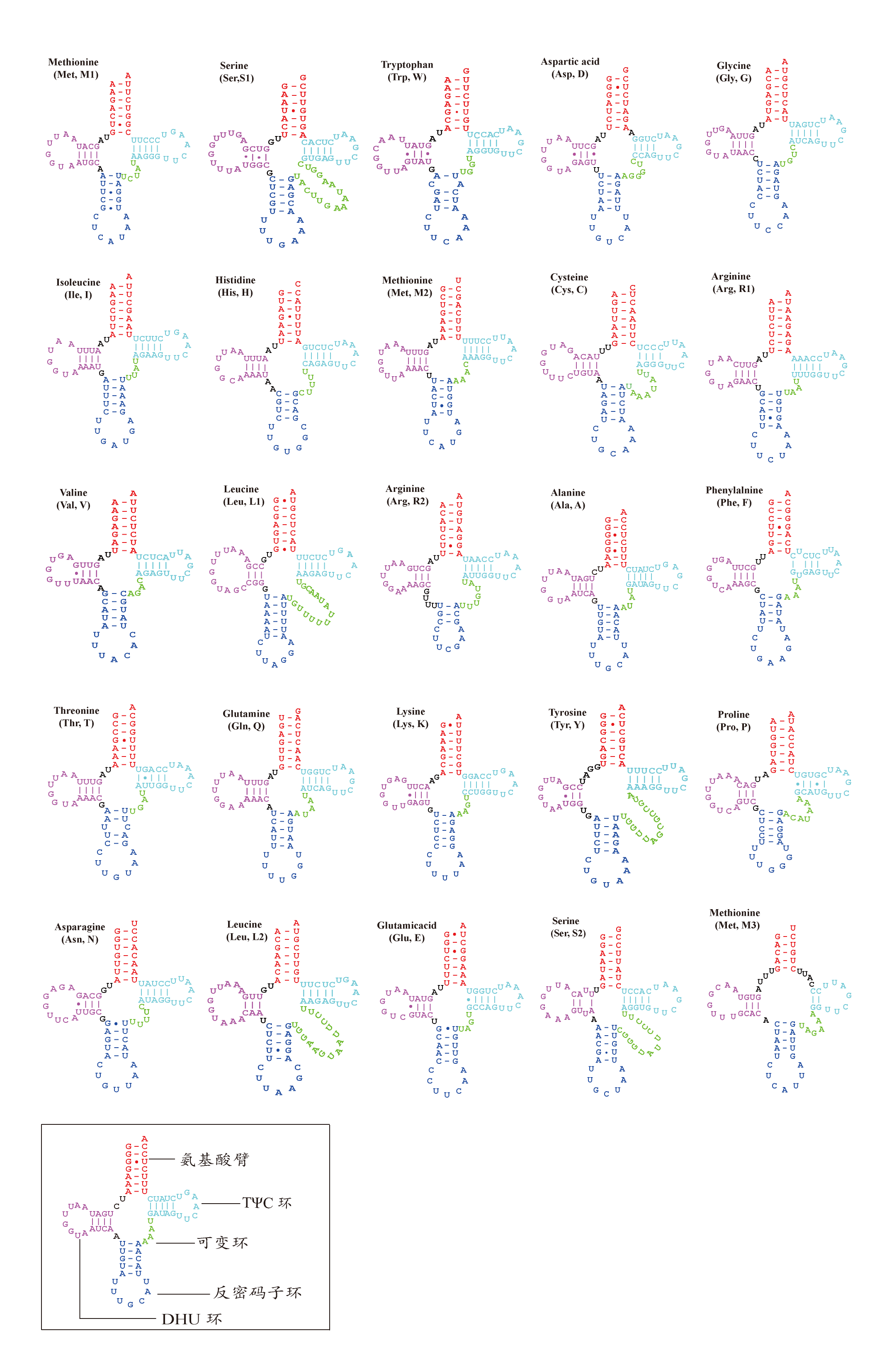

Supplement: SUPPLEMENTARY FIGURE S5 — R. hookeri tRNA Secondary Structures. [file Image_5.PNG]

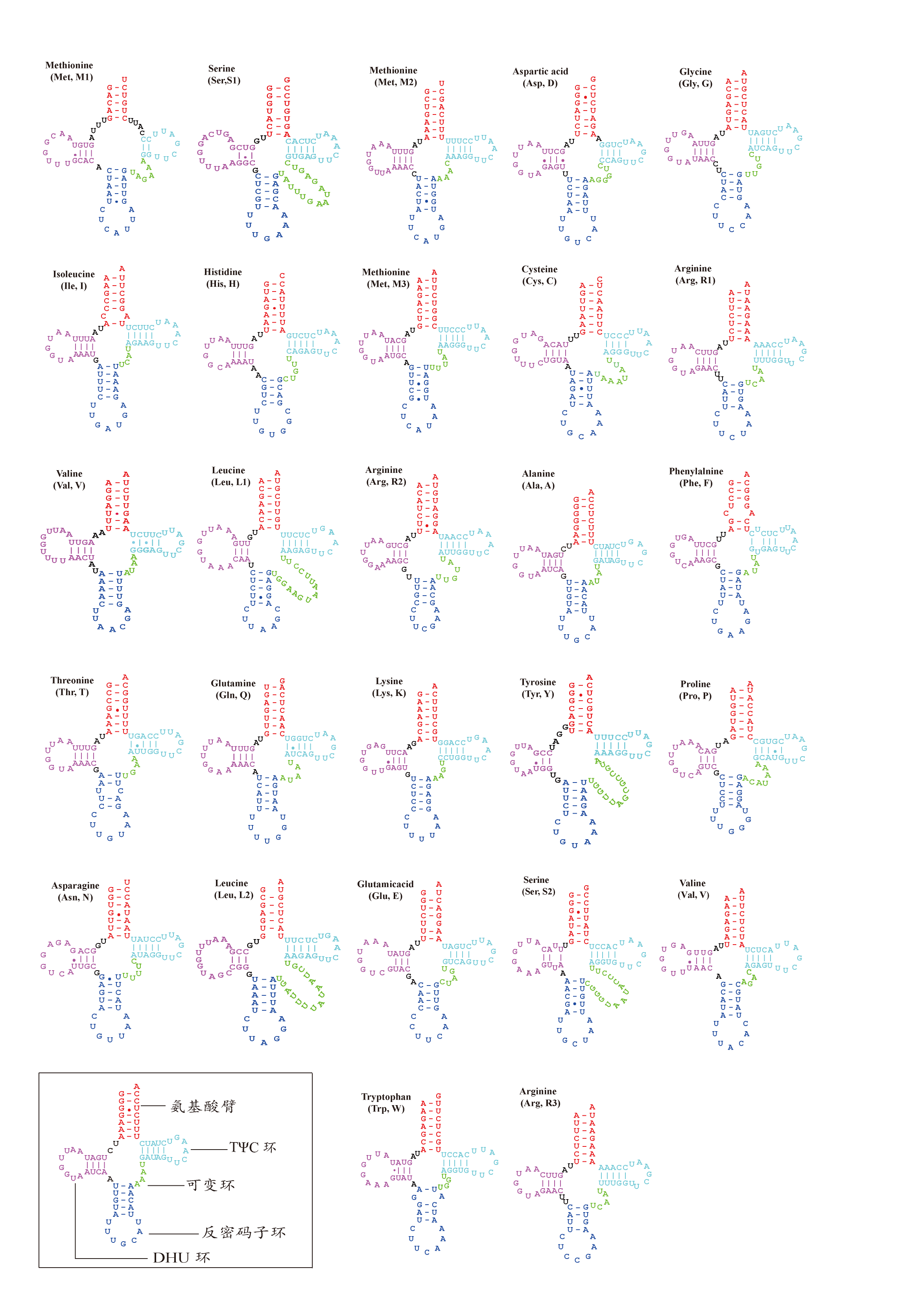

Supplement: SUPPLEMENTARY FIGURE S6 — R. aff. pelargonia tRNA Secondary Structures. [file Image_6.PNG]

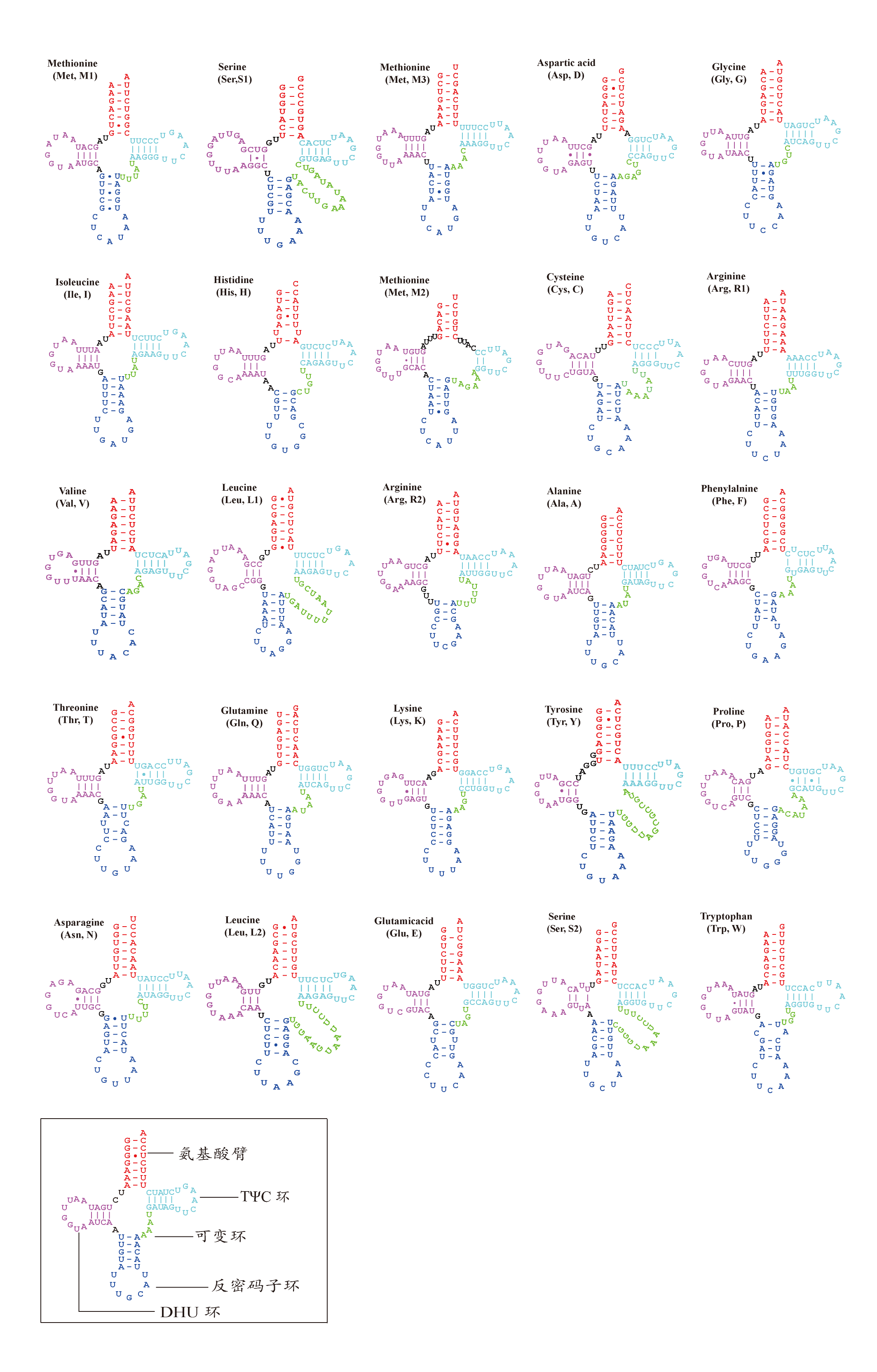

Supplement: SUPPLEMENTARY FIGURE S7 — R. sanguinea tRNA Secondary Structures. [file Image_7.PNG]

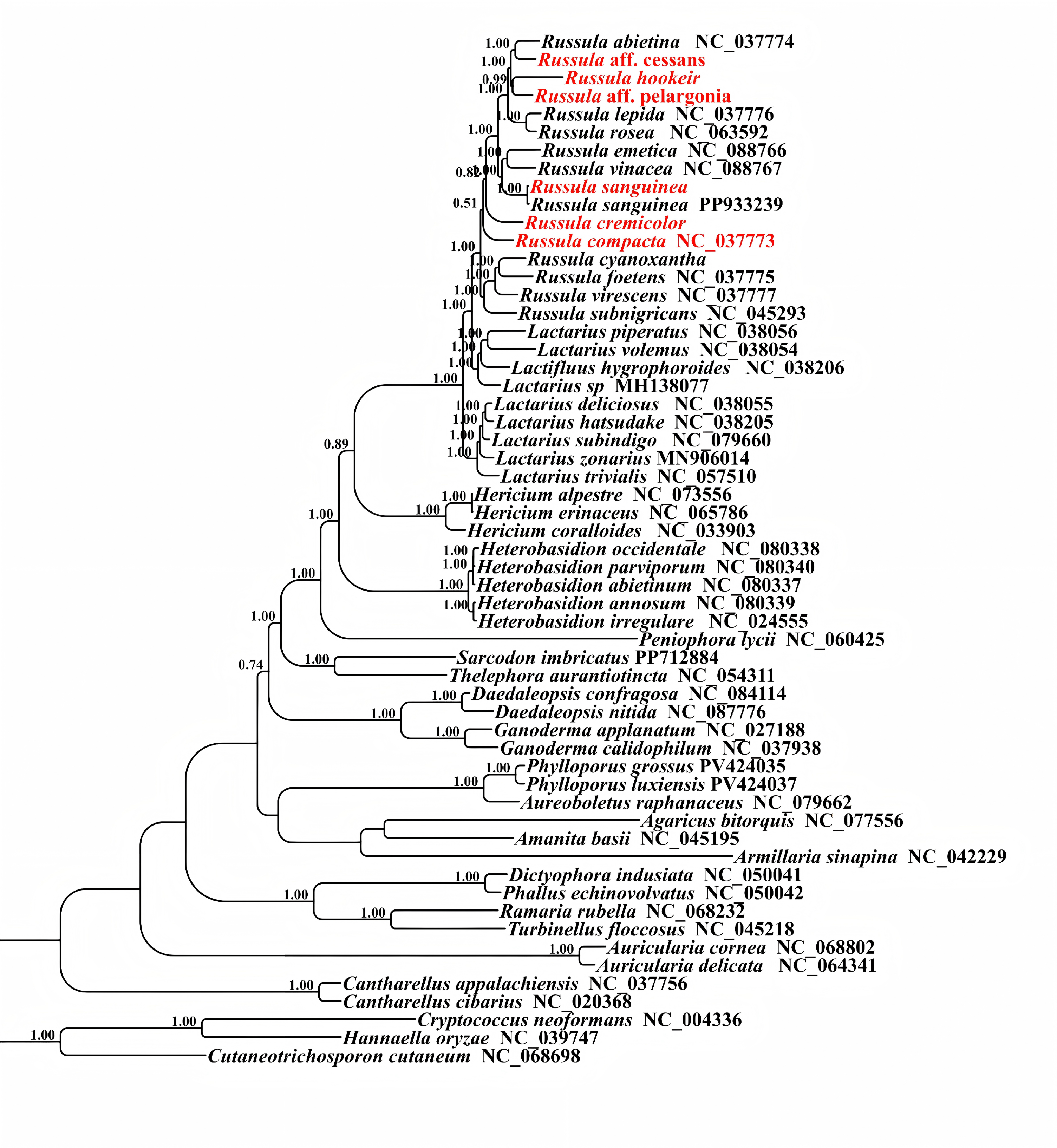

Supplement: SUPPLEMENTARY FIGURE S8 — Phylogenetic tree of Agaricomycetes inferred from the mitochondrial PCG dataset using BI. Newly sequenced Russula species are highlighted in red; nodal support values are shown as Bayesian posterior probabilities. [file Image_8.PNG]

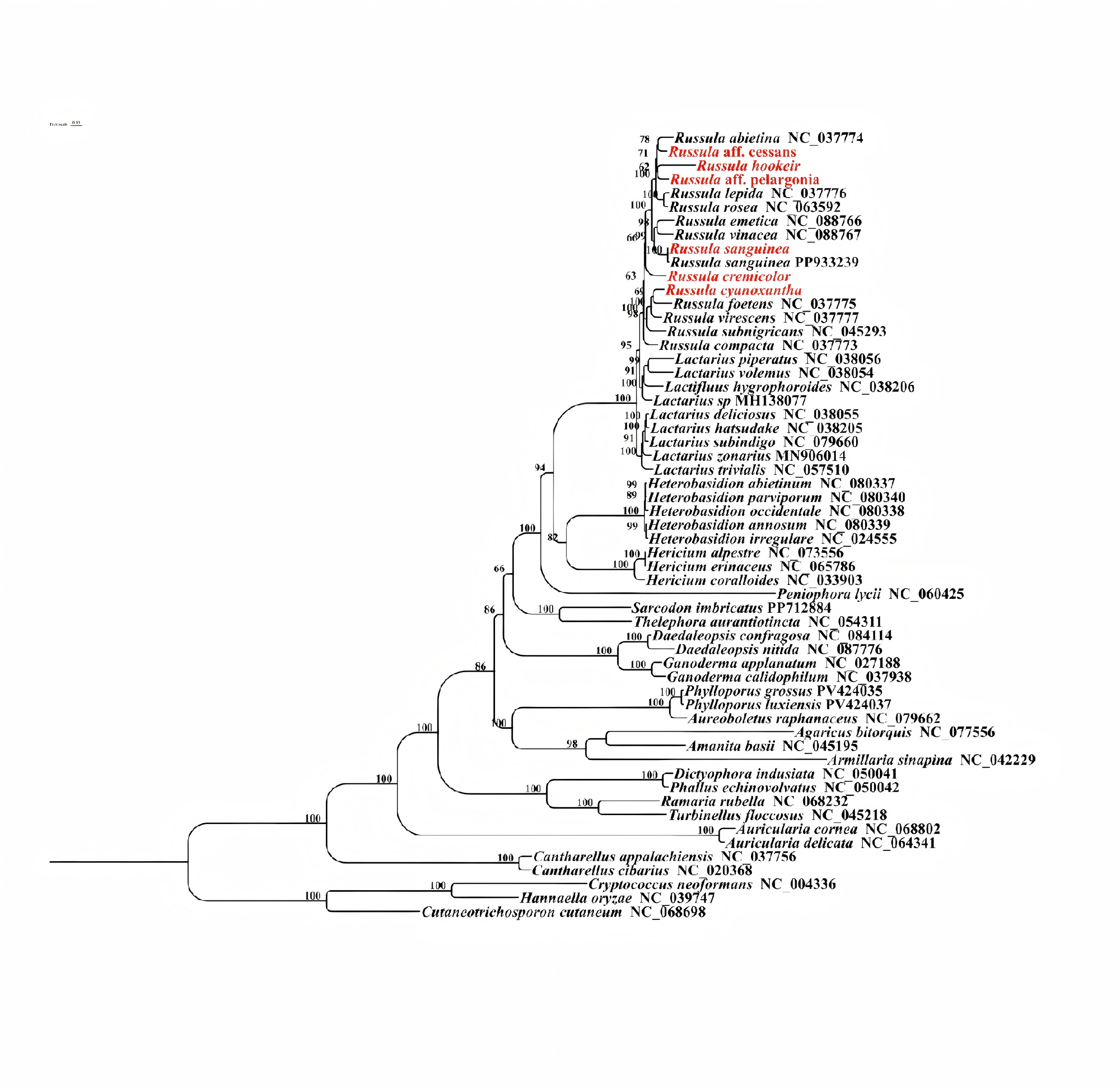

Supplement: SUPPLEMENTARY FIGURE S9 — Phylogenetic tree of Agaricomycetes inferred from the mitochondrial PCG12 dataset using ML. Newly sequenced Russula species are highlighted in red; nodal support values are shown as ML bootstrap values. [file Image_9.PNG]

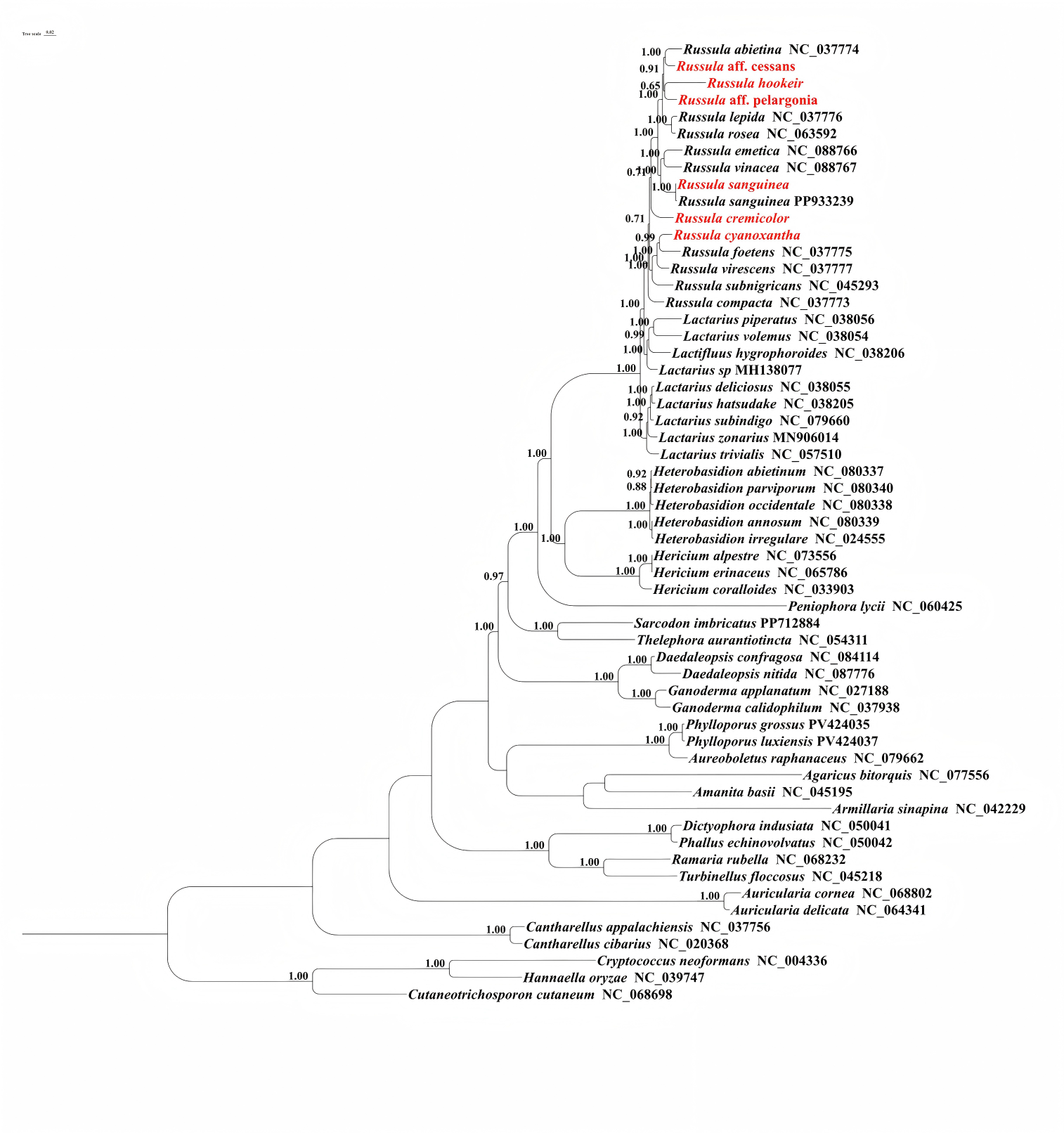

Supplement: SUPPLEMENTARY FIGURE S10 — Phylogenetic tree of Agaricomycetes inferred from the mitochondrial PCG12 dataset using BI. Newly sequenced Russula species are highlighted in red; nodal support values are shown as Bayesian posterior probabilities. [file Image_10.PNG]
