# Supplementary material for: Complete mitochondrial genomics reveals phylogenetic relationships and mitogenomic features in six ectomycorrhizal Russula species
Source: Front Microbiol. 2026 Jul 10;17:1865163. doi: 10.3389/fmicb.2026.1865163 (PMC13395875; doi:10.3389/fmicb.2026.1865163)
Supplement: Supplementary file 11 [file Table_1.docx]

**Supplementary Table S1.** Collect information of the six common ectomycorrhizal *Russula*

| Species name | Locality | Altitude (m) | Longitude and latitude | Habitat and distribution | Collection date | Voucher |
| --- | --- | --- | --- | --- | --- | --- |
| *Russula* aff. *cessans* | Qizhi Peak,  Panzhou City,  Guizhou Province | 2084 | 104°67′E, 25°55′N | Scattered in mixed forests dominated by Pinaceae | 7 August  2024 | MGBIII-20 |
| *Russula cremicolor* | Liangshui Valley,  Weining Yi and  Hui and Miao  Autonomous  County,  Guizhou  Province | 2222 | 103°89′E, 27°08′N | Solitary on Fagaceae and Pinaceae forest soils | 4 August  2024 | MGJIX-93 |
| *Russula cyanoxantha* | Huaxi University Town,  Huaxi District,  Guiyang City,  Guizhou Province | 1153 | 106°63′E, 26°37′N | Scattered on Fagaceae and Pinaceae forest soils | 23 June 2023 | M1030 |
| *Russula hookeri* | Magu Town,  Hezhang County,  Guizhou Province | 2374 | 104°64′E, 27°00′N | Solitary on Fagaceae forest soils | 4 August  2024 | MGJXI-022 |
| *Russula* aff. *pelargonia* | Huaxi University Town,  Huaxi District,  Guiyang City,  Guizhou Province | 1176 | 106°63′E, 26°37′N | Scattered on Fagaceae and Pinaceae forest soils | 23 June 2023 | M1011 |
| *Russula sanguinea* | Longjia Mountain  National Forest Park,  Longli County,  Guizhou Province | 1261 | 106°90′E, 26°46′N | Scattered in mixed forests dominated by Pinaceae | 3 June 2023 | M784 |
