# Supplementary material for: Complete mitochondrial genomics reveals phylogenetic relationships and mitogenomic features in six ectomycorrhizal Russula species
Source: Front Microbiol. 2026 Jul 10;17:1865163. doi: 10.3389/fmicb.2026.1865163 (PMC13395875; doi:10.3389/fmicb.2026.1865163)
Supplement: Supplementary file 12 [file Table_2.docx]

**Supplementary Table S2.** Specific Primer Sequences

| Primer Name | Specific Primer Sequence (5'→3') | Amplified Region | TM（℃） |
| --- | --- | --- | --- |
| Primer F | TCCATCTCACCCCTTTGTGC | ITS | 56.26 |
| Primer R | GTATCGCATTTCGCTGCGTT | ITS | 58.99 |
