# Supplementary material for: Complete mitochondrial genomics reveals phylogenetic relationships and mitogenomic features in six ectomycorrhizal Russula species
Source: Front Microbiol. 2026 Jul 10;17:1865163. doi: 10.3389/fmicb.2026.1865163 (PMC13395875; doi:10.3389/fmicb.2026.1865163)
Supplement: Supplementary file 13 [file Table_3.docx]

**Supplementary Table S3.** Key Mitogenomic Features of the Six *Russula* Species

| Species Name | GC % | GC Skew | | AT Skew | | Size(bp) | Number of tRNAs |
| --- | --- | --- | --- | --- | --- | --- | --- |
| *R.* aff. *cessans* | 22.400 | | 0.052 | | 0.006 | 57,924 | 25 |
| *R. cremicolor* | 23.000 | | 0.076 | | 0.011 | 48,621 | 26 |
| *R*. *cyanoxantha* | 22.200 | | 0.051 | | 0.003 | 46,307 | 26 |
| *R*. *hookeri* | 22.000 | | 0.074 | | -0.009 | 41,268 | 25 |
| *R.* aff. *pelargonia* | 21.800 | | 0.066 | | 0.001 | 56,617 | 27 |
| *R*. *sanguinea* | 22.100 | | 0.046 | | -0.012 | 49,462 | 25 |
